# Synthesis, Characterization, and Biological Evaluations of 1,3,5-Triazine Derivatives of Metformin Cyclization with Berberine and Magnolol in the Presence of Sodium Methylate

**DOI:** 10.3390/molecules22101752

**Published:** 2017-10-18

**Authors:** Han Cao, Shili Liao, Wenjing Zhong, Xuerong Xiao, Jiancheng Zhu, Weimin Li, Xia Wu, Yifan Feng

**Affiliations:** 1 Central Laboratory of Guangdong, Pharmaceutical University, Guangzhou 510000, China; caohan19930219@163.com (H.C.); shililiao_03@163.com (S.L.); ZWJzwj112@163.com (W.Z.); x1784502739@163.com (X.X.); edenss@foxmail.com (J.Z.); 2 College of Chinese Materia Medica, Guangzhou University of Chinese Medicine, Guangzhou 510000, China

**Keywords:** cyclization triazine, anti-inflammatory activity, anti-insulin resistance

## Abstract

The novel target products were synthesized in the formation of a triazine ring from berberine, magnolol, and metformin catalyzed by sodium methylate. The structures of products **1**–**3** were firstly confirmed by extensive spectroscopic analyses and single-crystal X-ray diffraction. The crystal structures of the target product **2** and the intermediate product **7b** were reported for the first time. All target products were evaluated for their anti-inflammatory and antidiabetic activities against INS-1 and RAW264.1 cells in vitro and all products showed excellent anti-inflammatory effects and anti-insulin resistance effects. Our studies indicated that new compounds **1**–**3** were found to be active against inflammation and insulin resistance.

## 1. Introduction

Berberine is an isoquinoline alkaloid originally isolated from the Chinese herb Coptis chinensis (Huang lian) and is one of the main components of R. Coptidis [1]. Magnolol is a 4-allyl-2-(5-allyl-2-hydroxy-phenyl) phenol, and is present in considerable quantities in the bark of the Houpu magnolia (Magnolia officinalis) [2]. Previous data showed that berberine and magnolol were extensively employed in traditional Chinese medicine for the treatment of diabetes, cancers, inflammations, and hyperlipoidemia [3,4,5,6,7,8].

Metformin has been widely applied in clinics since the 1950s [9]. Over the past decades, metformin has been recommended as a first-line antidiabetic therapy for the treatment of type 2 diabetes mellitus (T2DM) based on the official guidelines of the American Diabetes Association (ADA), the European Association for the Study of Diabetes (EASD), and the American Association of Clinical Endocrinologists (AACE) [10,11]. Moreover, it takes part in the regulation of reduced insulin resistance and blood glucose concentration [12].

According to a review of recent advances in structural modifications of metformin, a variety of metformin derivatives have been successfully synthesized. In 2005, Viollet synthesized new 3d and 4f complex compounds with metformin that were utilized for the treatment of diabetic patients with non-insulin-dependent diabetes mellitus [13]. Liu synthesized seven derivatives of 2-amino-4-dimethylamino-1,3,5-triazine and obtained pure target compounds from silica gel column chromatography in the same year [14]. Additionally, berberine and magnolol derivatives have likewise been synthesized. In 1995, K. Iwsav firstly synthesized berberrubine derivatives [15]. In 2010, Huang designed, synthesized, and evaluated a new series of berberine derivatives as AChE inhibitors [16]. In 2012, Khaing Zin synthesized clovanemagnolol from the reaction of magnolol and caryophyllene β-oxide, appending macromolecular groups onto the phenolic hydroxyl groups of magnolol for the first time [17].

However, these reports on berberine, magnolol, and metformin derivatives described individual structural transformations, while there are no reports on berberine, magnolol, and metformin combined in the formation of a triazine ring catalyzed by sodium methylate.

**Figure 1:**
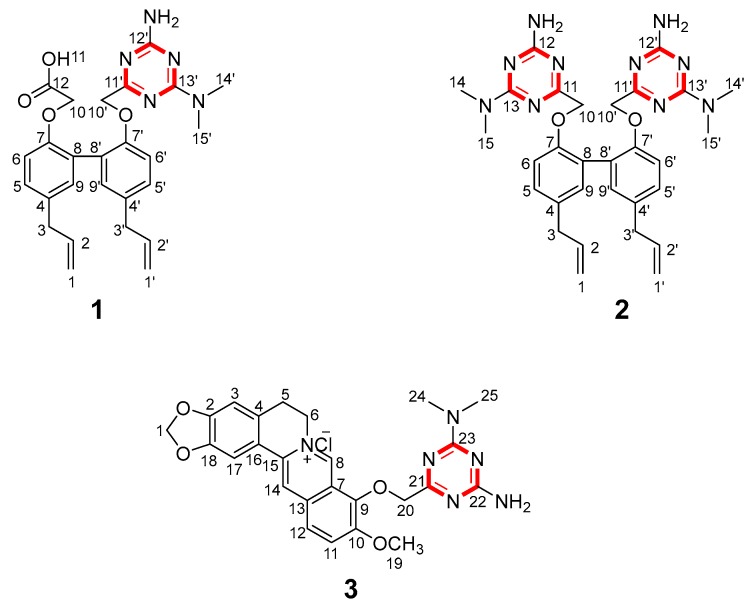
The structures of compounds **1**–**3**.

Our continuing interest in the chemistry of metformin, berberine, and magnolol and the absence of any reported synthetic approaches to the structurally distinct frameworks of products **1**–**3** (Figure 1) prompted us to begin investigations in this area. We synthesized compounds **1**–**3** combined in the formation of a triazine ring from berberine, magnolol, and metformin (published patents CN 106966997 A and CN 106928246 A) [18,19]. On this basis, we optimized the previous reaction conditions and identified their structure by UV, IR, ^1^H-NMR, ^13^C-NMR, 2D-NMR, and HRESIMS. In this study, the crystal structures of the target product **2** and the intermediate product **7b** were never reported in our cognition. The cytotoxic activities of these derivatives on INS-1 cells and RAW264.1 cells were established by the MTT method [20]. We also determined that PEG-2 and COX-2 released amounts of these compounds through ELISA assays, in order to investigate their potential biological and pharmacological activities.

## 2. Results and Discussion

### 2.1. Chemistry

**Scheme 1:**
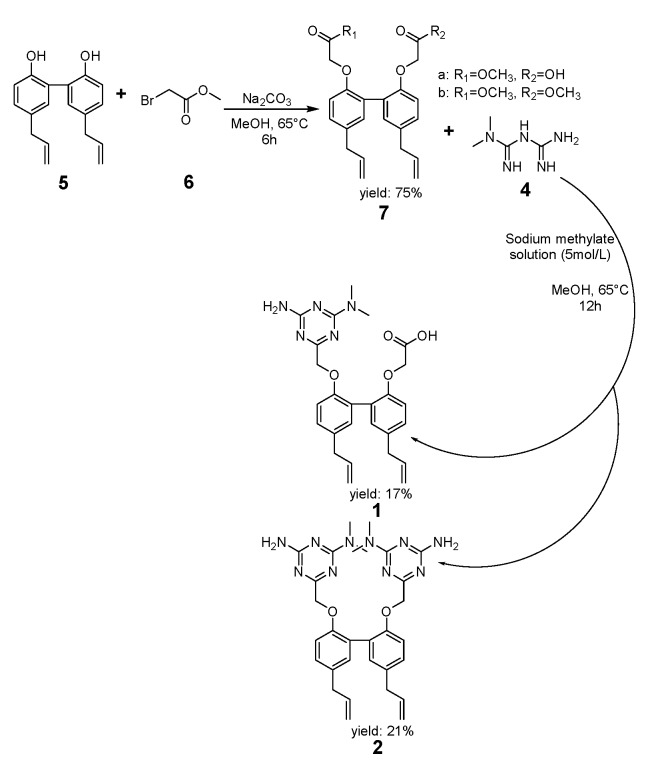
Cyclization of compounds **1** and **2**.

**Scheme 2:**
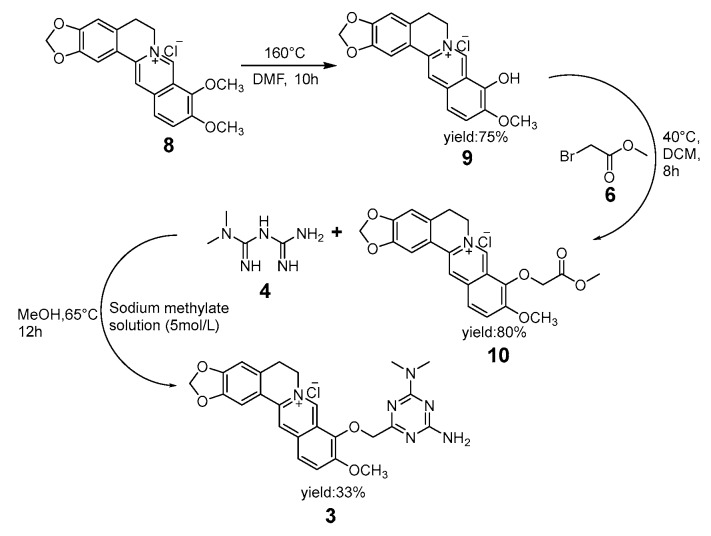
Cyclization of compound **3**.

The synthetic strategy for preparing compounds **1** and **2** is outlined in Scheme 1, and the strategy for compound **3** is outlined in Scheme 2. Briefly, it is based on the alkylation of phenolic OH groups of magnolol (**5**) and berberrubine (**9**) with methyl bromoacetate and a subsequent ring closure reaction with metformin (a biguanide) in alkaline medium to afford the final 1,3,5-triazine derivatives (**1**, **2** and **3**). During the development of compounds **1** and **2**, we started by alkylation; reacting magnolol (**5**) with methyl bromoacetate (**6**). The reaction proceeded smoothly under mild conditions. Two phenolic OH groups of magnolol (**5**) engaged in alkylation with the bromine group of methyl bromoacetate catalyzed by Na_2_CO_3_. Because the generated HBr was neutralized by Na_2_CO_3_, the alkylation reacting was more rapid and thorough, and compound **7** was found to be present in the form of esters. In order to obtain dissociated metformin, metformin hydrochloride and sodium methoxide were mixed in a solvent for 30 min at room temperature. In the process of mixing, the displacement reaction occurred between metformin hydrochloride and sodium methoxide. Because the solution of product **7** could participate in the subsequent reaction, product **7** was added dropwise to a cyclization reaction with metformin. Before the cyclization reaction, the ester groups of product **7** were hydrolyzed by sodium methoxide and the carbonyl groups on product **7** engaged in a ring closure reaction with the two imino groups of metformin (**4**) in the presence of sodium methylate to afford the final 1,3,5-triazine derivatives (**1** and **2**).

Compared with compounds **1** and **2**, the ring closure reaction of compound **3** underwent an analogous process. According to a review of Cao’s and Nechepurenko‘s studies [21,22], the initial step was a pintsch reaction in berberine hydrochloride (**8**), which led to the product berberrubine (**9**). Then, the hydroxyl radical of berberrubine (**9**) engaged in the nucleophile substitution reaction with the bromine group of methyl bromoacetate (**6**) and gave the intermediate product (**10**). With the similar cyclization process of products **1** and **2**, intermediate product **10** was stirred in the metformin solution, which consisted of metformin hydrochloride and sodium methoxide that had been mixed in a solvent for 30 min at room temperature and led to product **3**.

Then, we studied the reaction under various conditions of alkylation reactions (Tables S5 and S6). Our studies were initiated using dichloromethane (DCM) with Na_2_CO_3_ for compound **7**, methanol (MeOH) for compound **10**, and methanol (MeOH) with 8 mL of a sodium methylate (NaOCH_3_) solution (5 mol/L) for compounds **1**–**3** at room temperature (25 °C). After screening the reaction conditions, using Na_2_CO_3_ as the base catalyst and methanol as the solvent reacted at 65 °C for 6 h was determined to be optimal for the synthesis of product **7** (Table S5, entry 11). DCM as the solvent reacted at 45 °C for 8 h was determined to be optimal for the synthesis of product **10** in 80% yield (Table S6, entry 9).

Using these optimized conditions (Table S5, entry 11 and Table S6, entry 9), we examined the substrate scope of the cyclization reaction (Table S7). The best conditions for the reaction of compounds **1**–**3** were determined to be CH_3_ONa as the base catalyst and methanol as the solvent, reacted at 65 °C for 12 h (Table S7, entry 12).

### 2.2. Characterization of Compounds

**Figure 2:**
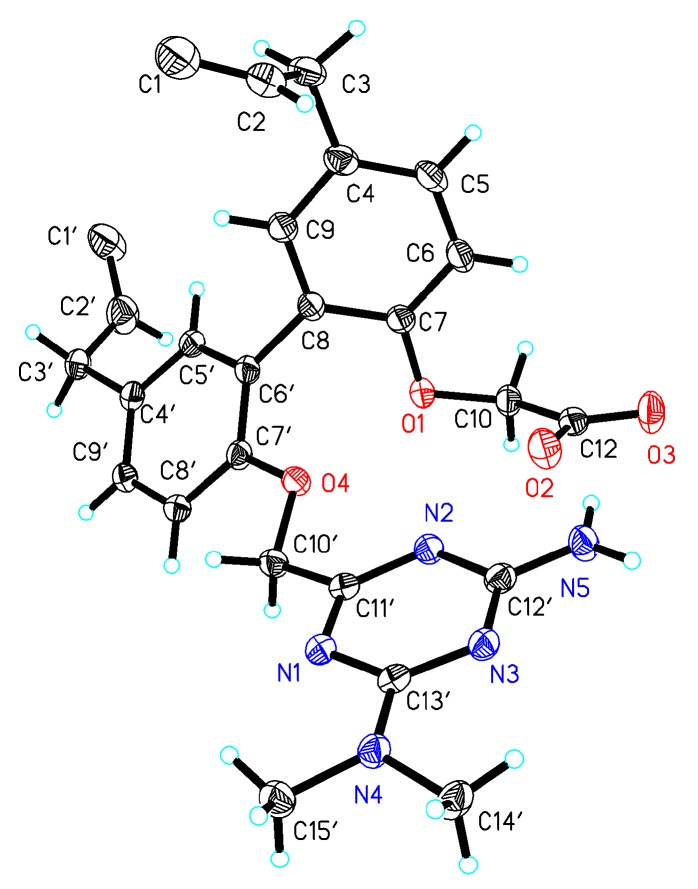
X-ray ORTEP (Oak Ridge Thermal Ellipsoid Plot) drawing of compound **1**.

**Figure 3:**
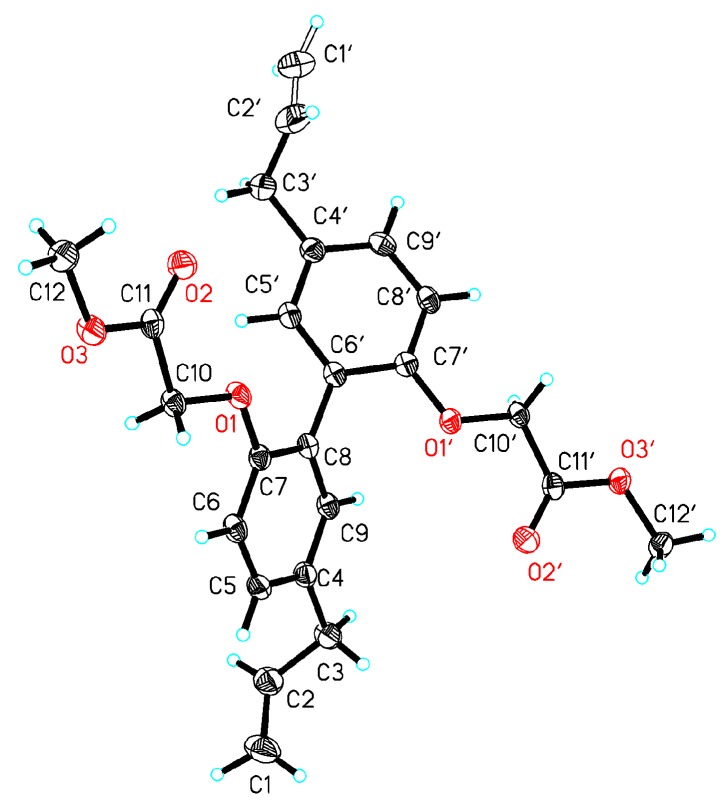
X-ray ORTEP (Oak Ridge Thermal Ellipsoid Plot) drawing of coampound **7b**.

**Figure 4:**
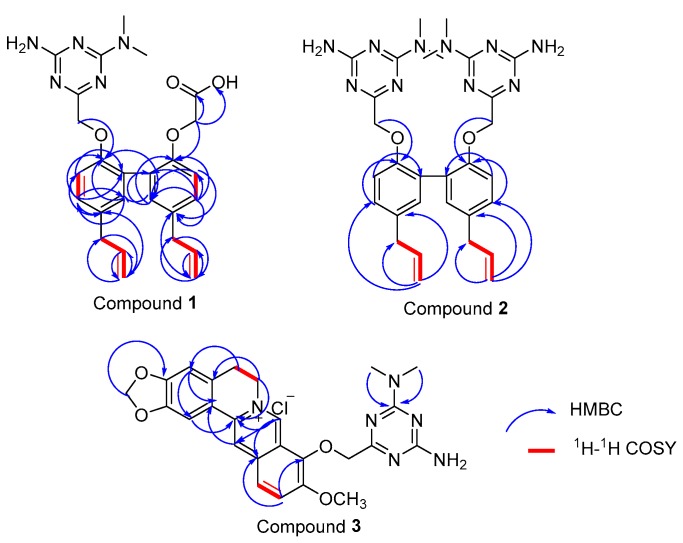
Key heteronuclear multiple bond correlation (HMBC) and ^1^H-^1^H COSY correlations of compounds **1**–**3**.

Target compound **1** was confirmed to possess a molecular formula of C_26_H_29_N_5_O_4_ by high-resolution electrospray ionization mass spectrometry (HRESIMS) (*m*/*z* = 476.2322 M^+^, calcd = 476.2298) and ^13^C-NMR data, requiring 13 degrees of unsaturation. Compound **2** was confirmed to possess a molecular formula of C_30_H_36_N_10_O_2_ by HRESIMS (*m*/*z* = 569.3101 M^+^, calcd = 569.3101) and ^13^C-NMR data. The Fourier transform infrared (FT-IR) spectrum of **1** (Figure S7) suggested the presence of a hydroxyl group (3225.36 cm^−1^), a methyl group (2929.34 cm^−1^), a carbonyl group (1680.66 cm^−1^), and benzene rings (1571.7 cm^−1^ and 1630.52 cm^−1^). A comparison of the FT-IR data of **2** and **1** (Figures S15 and S7) suggested that these compounds shared the same methyl group and benzene rings. The ^13^C-NMR and ^1^H-NMR spectra of **1** clearly showed the two methyl groups located on the N atom (Tables S8 and S9). A detailed examination of the 2D-NMR spectra of **1** confirmed the existence of two benzene rings—ring-A and ring-B—and the triazine ring-C. The heteronuclear multiple bond correlation (HMBC) of H-5/C-7, C-9, C-8; H-6/C-4, C-9; H-5′/C-7′, C-9′; and H-8′/C-6′, C-5′, C-4′, combined with the ^1^H-^1^H correlation spectroscopy (COSY) correlations of H-5/H-6, clearly showing the locations of the benzene ring-A and -B. The triazine ring-C was established based on the HMBC correlations of C-13′/H-14′, H-15′ and H-10′/C-11′, C-12′. The relative configurations of compounds **1** and **7** were determined by X-ray diffractions using Cu-Kα radiation with a refinement parameter of 0.04. The crystal structure of compound **1** is deposited in the CCDC as number 1,547,785 (Figure 2), while compound **7b** is 1,547,784 (Figure 3). Furthermore, the HMBC and ^1^H-^1^H COSY correlations are shown in Figure 4.

Compound **2** has a completely symmetric structure. A detailed comparison of the ^13^C-NMR data (Table S10) and 2D-NMR spectra of **2** and **1** revealed that the structure of **2** was very similar to that of **1**. The only difference was the replacement of the carbonyl group in **1** by an additional moiety of **4** in **2**, as confirmed by the HMBC of H-10/C-11, H-14/C-13, and H-15/C-13.

The molecular formula of compound **3**, C_25_H_25_N_6_O_4_^+^, was established by HRESIMS (*m*/*z* = 473.1936 M^+^, calcd = 473.1937) and ^13^C-NMR data, requiring 13 degrees of unsaturation. The FT-IR spectrum of compound **1** (Figure S23) suggested the presence of a primary amine group (1674.87 cm^−1^) and benzene rings (1600.63 cm^−1^, 1553.38 cm^−1^, and 1491.67 cm^−1^). The ^1^H-NMR data of **3** displayed signals for two methyl groups at δH = 2.179 (d) and a methoxyl group at δH = 3.10 (s) (Figure S17). The HMBC of H-3/2-C, 17-C, 16-C; 1-H/2-C; and 17H/15-C indicated the existence of benzene ring-A. Ring-B contained an N atom between C-5 and C-15, as confirmed from the HMBC of 5-H/4-C, 6-C and 6-H/5-C, 15-C, combined with the ^1^H-^1^H COSY correlations of H-5/H-6. Meanwhile, ring-C was deduced by the HMBC of 8H/13C, 7C, 15C, 14C and 14H/8C, 15C, 16C. The HMBC of 11H/9C, 13C, 12C, 10C and 12H/13C, 10C, 11C, combined with the ^1^H-^1^H COSY correlations of 12H/11H, elucidated the structure of ring-D. The HMBC of 20H/21C; 24H/22C, 23C; and 25H/22C, 23C suggested the presence of the triazine ring.

### 2.3. Biological Evaluation

**Figure 5:**
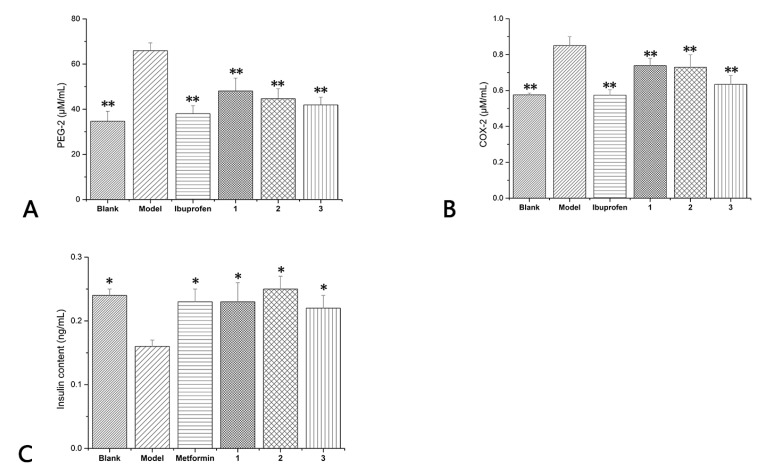
(**A**,**B**) The levels of COX-2 and PEG-2 which influenced by compounds **1**–**3** and ibuprofen. The blank group was not stimulated by LPS. The model group and compound **1**–**3** groups were stimulated by LPS. * *p* < 0.05, ** *p* < 0.01 compared to the model; (**C**) Glucose-mediated (16.7 mM) insulin secretion by INS-1 cells incubated in the presence of compounds **1**–**3**. The blank group was not stimulated by culture medium containing 40 mmol/L glucose. The model group and compound **1**–**3** groups were stimulated by culture medium containing 40 mmol/L glucose. * *p* < 0.05, ** *p* < 0.01 compared to the model.

In order to evaluate the anti-inflammatory activities and antidiabetic activities of the synthesized compounds **1**–**3**, we chose RAW264.1 cells (mice inflammatory cells) and INS-1 cells (mouse insulinoma cells). Cell viability was measured by using 3-(4,5-dimethylthiazol-2-yl)-2,5-diphenyl tetrazolium bromide (MTT) assay. RAW264.7 cell cultures were pretreated with a series of compounds **1**–**3** or vehicle to evaluate the effects of compounds **1**–**3** on the release of COX-2/PEG-2. Two target cytokines were markedly increased in LPS (Lipopolysaccharide)-stimulated RAW264.7 cells; the increase was dramatically diminished by compounds **1**, **2** at 10 µMol/L and compound **3** at 25 µMol/L concentrations. The level of COX-2 was decreased by 13.06%, 14.24%, and 25.41%, respectively, by compounds **1**–**3**, and the level of PEG-2 was decreased by 27.05%, 32.26%, and 36.41%. Ibuprofen was used as positive control and the level of COX-2/PEG-2 was decreased by 32.82% and 42.31% (Figure 5A,B). In general, compounds **1**–**3** all performed remarkable anti-inflammatory activity.

Compounds **1**–**3** were assessed for their effects on the insulin secretion of rat pancreatic INS-1 β-cells. All the products had a significant stimulatory effect on the insulin secretion from INS-1 cells. In the presence of compound **3**, an increase of 143% in insulin release was recorded compared to the model whereas increases of 156% and 137% were recorded in the presence of compound **2** and compound **1**, respectively (Figure 5C). Metformin was used as positive control and insulin increased 147% compared with the model. The results showed that all compounds had excellent antidiabetic effects and stimulatory effects on the insulin secretion from INS-1 cells, and the effects of compound **2** exceeded that of metformin.

## 3. Materials and Methods

### 3.1. General Experimental Procedures

FT-IR spectra were recorded on a Jasco FT-IR-4600 microscopic spectrometer (Jasco Corporation, Tokyo, Japan). UV spectra were measured on a Jasco V-550 UV/Vis spectrophotometer (Jasco Corporation, Tokyo, Japan). Melting points were determined using a Beijing Tech X-6 microscopic melting point apparatus (Beijing Tech Corporation, Beijing, China). The 1D- and 2D-NMR spectra were taken on a Bruker-AVANCEIII-010601AM-500 spectrometer (Bruker Corporation, Karlsruhe, Germany) using TMS (Tetramethylsilane) as an internal standard. ESIMS was measured on a Waters UPLC/Q-TOF mass spectrometer (Waters Corporation, Milford, MA, USA). The X-ray crystallographic data were obtained on a Rigaku X-ray diffractometer (Rigaku Corporation, Tokyo, Japan) using Cu-Kα radiation. HPLC was performed on a Waters 2695 liquid chromatograph (Waters Corporation, Milford, MA, USA) with DIKMA-Diamonsil-C18 (5 μm, 200 × 4.6 mm). Reactions were monitored by HPLC (DIKMA-Diamonsil-C18, 5 μm, 200 × 4.6 mm).

### 3.2. Synthesis and Purification of Compounds ***1***–***3***

All the starting reactants, solvents, and catalysts were commercially available unless otherwise stated. All solvents, except for the aqueous media, were purified by standard techniques to exclude moisture.

#### 3.2.1. Synthesis of Compound **7**

Magnolol (2.66 g, 0.01 mol), methyl bromoacetate (2 mL, 0.02 mol), and anhydrous potassium carbonate (Na_2_CO_3_) (0.02 mol) in absolute methanol (50 mL) were reacted at 65 °C for 6 h. After the completion of the reaction, the mixture was filtrated while hot and the methanol was removed by vacuum distillation to give a solid residue (2.32 g, 86%, containing a mixture of 36% **7a** and 46% **7b**). Crystals of pure **7b** could be separated manually and were used for characterization.

*Dimethyl 2,2′-{[5,5′-diallyl-(1,1′-biphenyl)-2,2′-diyl]bis(oxy)}diacetate* (**7b**): White powder; m.p. 209–211 °C; Yield: 75%; ESI-MS *m*/*z* 409.17 M^−^; ^1^H-NMR (500 MHz, DMSO-*d*_6_) δ ppm: 3.31 (4H, d, *J* = 7.0 Hz, H-1, H-1′), 3.65 (6H, s, H-12, H-12′), 4.67 (4H, s, H-10, H-10′), 5.01–5.11 (2H, m, H-3, H-3′), 5.91–5.99 (2H, m, H-2, H-2′), 6.89 (2H, d, H-9, H-9′), 7.04 (2H, d, *J* = 2.5 Hz, H-6, H-6′), 7.04–7.10 (2H, m, H-5. H-5′); ^13^C-NMR (125 MHz, DMS0-*d*_6_) δ ppm: 38.5 (C, C-1, C-1′), 51.6 (C, C-12, C-12′), 65.4 (C, C-10, C-10′), 112.5 (C, C-9, C-9′), 115.5 (C, C-3, C-3′), 127.3 (C, C-8, C-8′), 128.2 (C, C-5, C-5′), 131.3 (C, C-6, C-6′), 132.1 (C, C-4, C-4′), 137.8 (C, C-2, C-2′), 153.5 (C, C-7, C-7′), 169.3 (C, C-11, C-11′).

#### 3.2.2. Synthesis of Compounds **1** and **2**

Metformin hydrochloride (6.6 g, 0.04 mol), a sodium methylate solution (5 mol/L, 8 mL), and absolute methanol (20 mL) were added to a flask and mixed at room temperature for 30 min. A methanol solution of compound **7** (4.4 g, 0.01 mol, 50 mL) was added dropwise to the flask at 65 °C, and the mixture was reacted for 12 h. After the reaction, the mixture was filtrated and the precipitate was isolated (5.33 g, **1**: 47.85%, **2**: 29.79%). Methanol (70 mL) and the precipitate were mixed at 60 °C for 6 h and then filtered to obtain a mixture (3.34 g, **1**: 56.89%, **2**: 33.58%). Then, the mixture and 70 mL DCM were mixed at 40 °C for 4 h and then filtered to obtain dichloromethane solution, which was vacuum distilled to eliminate the DCM. The white powder was washed with water to obtain compound **2** (1 g, 0.0017 mol, 97%). Then, absolute methanol solution (26 mL) of the filter cake was added into formic acid (1 mL) and then precipitated in 4 °C for 12 h. Then, the mixture was filtrated and the precipitated was isolated (**1**, 0.99 g, 0.0021 mol, 97%).

*2-{{5,5′-Diallyl-2′-{[4-amino-6-(dimethylamino)-1,3,5-triazin-2-yl]methoxy}-(1,1′-biphenyl)-2-yl}oxy}acetic acid* (**1**): White powder; m.p. 277–280 °C; Yield: 17%; ESI-MS *m*/*z* 476.2322 M^+^; ^1^H-NMR (500 MHz, DMSO-*d*_6_) δ ppm: 3.30 (4H, t, *J* = 7.5 Hz, H-1, H-1′), 5.88–5.99 (2H, m, H-2, H-2′), 4.98–5.04 (2H, m, H-3), 5.04–5.10 (2H, m, H-3′), 6.90 (1H, d, *J* = 8.4 Hz, H-5), 7.02–7.11 (4H, m, H-5′, H-6, H-8′, H-9), 6.86 (1H, d, *J* = 8.4 Hz, H-9′), 4.69 (2H, s, H-10), 4.55 (2H, s, H-10′), 2.99 (6H, s, H-14′, H-15′), 6.80 (2H, s, H-16′); ^13^C-NMR (125 MHz, DMSO-*d_6_*) δ ppm: 39.6 (C, C-1, C-1′), 137.9 (C, C-2, C-2′), 115.4 (C, C-3, C-3′), 131.6 (C, C-4), 131.5 (C, C-4′), 112.8 (C, C-5), 131.3 (C, C-5′), 127.4 (C, C-6), 127.1 (C, C-6′), 154.4 (C, C-7), 153.8 (C, C-7′), 127.1 (C, C-8), 127.4 (C, C-8′), 131.2 (C, C-9), 112.5 (C, C-9′), 70.4 (C, C-10), 65.6 (C, C-10′), 170.5 (C, C-11′), 172.8 (C, C-12), 166.5 (C, C-12′), 165.1 (C, C-13′), 35.6 (C, C-14′).

*6,6-{[5,5′-Diallyl-(1,1′-biphenyl)-2,2′-diyl]bis(methylene)}bis(N2,N2-dimethyl-1,3,5-triazine-2,4-diamine)* (**2**): White powder; m.p. 211–213 °C; Yield: 21%; ESI-MS *m*/*z* 569.3101 M^+^; ^1^H-NMR (500 MHz, DMSO-*d*_6_) δ ppm: 3.28 (4H, t, *J* = 6.7 Hz, H-1, H-1′), 5.90 (2H, ddt, *J* = 16.8, 10.0, 6.8 Hz, H-2, H-2′), 5.05 (2H, s, H-3), 4.96 (2H, s, H-3′), 7.01, 7.02 (2H, dd, *J* = 8.4, 2.3 Hz, H-5, H-5′), 7.11 (2H, d, *J* = 2.3 Hz, H-6, H-8′), 6.92 (2H, d, *J* = 8.5 Hz, H-9, H-9′), 4.71 (4H, S, H-10, H-10′), 2.99 (12H, s, H-15, H-16, H-15′, H-16′), 6.8 (4H, s, H-NH_2_, NH_2_′), 4.69 (2H, s, H-10), 4.55 (2H, s, H-10′), 2.99 (6H, s, H-14′, H-15′), 6.80 (2H, s, H-16′); ^13^C-NMR (125 MHz, DMSO-*d*_6_) δ ppm: 38.6 (C, C-1, C-1′), 137.9 (C, C-2, C-2′), 115.3 (C, C-3, C-3′), 131.4 (C, C-4, C-4′), 127.2 (C, C-5, C-5′), 131.1 (C, C-6, C-6′), 154.4 (C, C-7, C-7′), 127.9 (C, C-8, C-8′), 112.9 (C, C-9, C-9′), 70.5 (C, C-10, C-10′), 172.8 (C, C-11, C-11′), 166.5 (C, C-12, C-12′), 165.1 (C, C-13, C-13′), 35.6 (C, C-14, C-14′, C-15, C15′).

#### 3.2.3. Synthesis of Compound **9**

Berberine (3.71 g, 0.01 mol) in DMF (20 mL) was reacted at 160 °C for 2 h. After the reaction, the mixture was then poured into cold water and a red precipitate was generated. The red solid was filtrated (2.78 g, 94%).

*10-DiMethoxy-5,6-dihydro-(1,3)dioxolo(4,5-g)isoquinolino(3,2-a)isoquinolin-7-ium chloride hydrate* (**9**): Red crystal; m.p. 245 °C; Yield: 75%; ESI-MS *m*/*z* 322.1002 M^+^; ^1^H-NMR (500 MHz, DMSO-*d*_6_) δ ppm: 9.07 (s, 1H, H-5), 7.97 (s, 1H, H-14), 7.60 (s, 1H, H-17), 7.20 (d, *J* = 8 Hz, 1H), 6.94 (s, 1H, H-3), 6.34 (d, *J* = 8.5 Hz, 1H, H-12), 6.08 (s, 2H, H-1), 4.47 (s, 2H, H-6), 3.72 (s, 3H), 3.03 (d, 2H, H-5); ^13^C-NMR (125 MHz, DMSO-*d*_6_) δ ppm: 167.1, 149.6, 148.3, 147.2, 145.7, 133.2, 132.0, 129.2, 121.8, 121.31, 119.9, 117.1, 108.2, 104.6, 101.5, 104.6, 101.5, 100.8, 55.7, 52.3, 27.4.

#### 3.2.4. Synthesis of Compound **10**

Berberrubine (3.57 g, 0.01 mol) and methyl bromoacetate (4 mL, 0.04 mol) were reacted in DCM (20 mL) at 45 °C for 8 h. After the reaction, the mixture was filtered, and the precipitate was isolated. Ethanol (95%) was added, and the solution was stirred and refluxed for 4 h at 70 °C to obtain compound **10** (3.15 g, 0.008 mol, 4%).

*10-Methoxy-9-(2-oxoethoxy)-(1,3) dioxolo (4,5-g) isoquinolino (3,2-a) isoquinolin-7-ium chloride* (**10**): Yellow powder; m.p. 212–215 °C; Yield: 80%; ESI-MS *m*/*z* 395.11 M^+^; ^1^H-NMR (500 MHz,DMSO-*d*_6_) δ ppm: 9.9 (s, 1H), 8.9 (s, 1H), 8.20 (d, *J* = 9.2 Hz, 1H), 8.00 (d, *J* = 9.1 Hz, 1H), 7.80 (s, 1H), 7.10 (s, 1H), 6.18 (s, 2H), 5.07 (s, 2H), 4.94 (t, *J* = 6.3 Hz, 2H), 4.03 (s, 3H, -OCH_3_), 3.72 (s, 3H, -OCH_3_), 3.23–3.15 (m, 2H); ^13^C-NMR (125 MHz, DMSO-*d*_6_) δ ppm: 169.3, 149.8, 149.2, 147.6, 145.7, 141.4, 137.5, 132.9, 130.6, 126.7, 123.5, 121.1, 120.4, 120.1, 108.4, 105.4, 102.1, 69.2, 57.1, 55.4, 51.8, 26.3.

#### 3.2.5. Synthesis of Compound **3**

Metformin hydrochloride (4.125 g, 0.025 mol), a sodium methylate solution (5 mol/L, 8 mL), and absolute methanol (60 mL) were added to a flask and mixed at room temperature for 30 min. The absolute methanol (15 mL) solution of compound **10** (1.67 g, 0.004 mol, 94%) was added dropwise to the flask at 65 °C, and the resulting mixture was reacted for 12 h. After the reaction, the mixture was filtered, and the precipitate was isolated (1.78 g, 36.25%). Methanol (100 mL) and the precipitate were mixed at 60 °C until the filtrate become colorless, and then the mixture was filtrated again (1.25 g, 68.17%). Compound **3** was obtained from the precipitate (0.62 g, 0.0013 mol, 97%) after crystallization in ethanol (3.6 mL) and concentrated hydrochloric acid (22 μL).

*9-[4-Amino-6-(dimethylamino)-1,3,5-triazin-2-yl]-10-methoxy-(4,5-g)isoquinolino(3,2-a)isoquinolin-7-ium chloride* (**3**): Red solid; m.p. 215–217 °C; Yield: 33%; ESI-MS *m*/*z* 473.1936 M^+^; ^1^H-NMR (500 MHz, DCOOD-*d*_2_) δ ppm: 7.17 (2H, s, H-1), 5.84 (1H, s, H-3), 2.17 (2H, s, H-5), 3.83–4.02 (2H, m, H-6), 6.41 (1H, s, H-8), 6.74 (1H, d, *J* = 8.5 Hz, H-11), 6.94 (1H, d, *J* =8 .5 Hz, H-12), 5.48 (1H, s, H-14), 5.48 (1H, s, H-17), 3.10 (3H, s, H19), 5.02 (2H, s, H-20), 2.21 (4H, s, H-24, H-25); ^13^C-NMR (125 MHz, DCOOD-*d*_2_) δ ppm: 166.5 (C, C-1), 121.2 (C, C-2), 102.4 (C, C-3), 159.7 (C, C-4), 27.1 (C, C-5), 53.7 (C, C-6), 130.9 (C, C-7), 106.4 (C, C-8), 123.9 (C, C-9), 142.1 (C, C-10), 120.5 (C, C-11), 132.7 (C, C-12), 131.1 (C, C-13), 108.9 (C, C-14), 143.0 (C, C-15), 151.3 (C, C-16), 147.7 (C, C-17), 161.4 (C, C-18), 58.3 (C, C-19), 103.0 (C, C-20), 148.9 (C, C-21), 156.4 (C, C-22), 156.0 (C, C-23), 38.1 (C, C-24), 37.9 (C, C-25).

### 3.3. HPLC Analysis of the Products

The yields of products were determined by HPLC analysis on the crude reaction mixture. The analytical HPLC system consisted of a Waters 2695 liquid chromatograph, a Waters 2996-visible detector, and Empower chromatography data station software. All samples were analyzed by HPLC using a reverse-phase DIKMA-Diamonsil-C18 (5 μm, 200 × 4.6 mm) at room temperature with a flow rate of 1.0 mL/min. For products **1** and **2**, the mobile phases consisted of solvent A (0.2% phosphoric acid in water, *v*/*v*) and solvent B (acetonitrile). The gradient elution was as follows: 45% B and 55% A. The flow rate was set at 1.0 mL/min. The sample injection volume was 10 µL. The UV detector was set at 277 nm. For product **3**, the gradient elution was as follows: initially, 0–50 min, 10–50% B; 50–55 min, 50–10% B; 55–60 min, 10% B. The flow rate was set at 1.0 mL/min. The sample injection volume was 10 µL. The UV detector was set at 254 nm.

### 3.4. Cellular Proliferation Assay

#### 3.4.1. Testing the Cytotoxicities of **1**–**3** against Mouse Macrophages and Mouse Insulinoma

Compounds **1**–**3** were tested for their cytotoxicities against RAW264.7 mouse macrophages and INS-1 cell lines. RAW264.7 cells were cultured in DMEM, and INS-1 cells were cultured in RMPI 1640. All cell lines were supplemented with 10% fetal bovine serum (FBS, Gibco Laboratories, Grand Island, NY, USA), 100 IU/mL penicillin, and 100 µg/mL streptomycin at 37 °C with 5% CO_2_. The media were changed every day. The viabilities of cells were determined by an MTT colorimetric assay system. For the toxicity assay, RAW264.7 and INS-1 cells were trypsinized and seeded onto 96-well plates at a density of 1 × 10^4^ cells per well. Then, the cells were treated with various concentrations (5, 10, 25, 50, 100, 200, 400, 800, and 1600 μM) of compounds **1**–**3** with a volume of 100 μL in sextuplicates. After 48 h of treatment, 20 μL of MTT (5 mg/mL) was added to each well, and the plates were incubated for 4 h at 37 °C. Then, DMSO (100 μL/well) was added. The optical density of lysate at 490 nm was measured by an enzyme immunoassay instrument (SpectraMax Plus 384, Molecular Devices, Sunnyvale, CA, USA).

#### 3.4.2. Testing the Anti-Inflammatory Activities of **1**–**3** against a Mouse Insulinoma Cell Line

For the anti-inflammatory activity assay, RAW264.7 cells were trypsinized and seeded onto 96-well plates at a density of 1 × 10^4^ cells per well. Then, according to the results of the cytotoxicities of **1**–**3** against mouse macrophages and mouse insulinoma, we chose cells whose survival rate was between 80–90% and the cells were treated with the target compounds (10 μM for compounds **1** and **2**, 25 μM for compound **3**) with a volume of 100 μL in sextuplicates, and ibuprofen (Sigma Inc., Shenzhen, China) was used as a positive control. After 1 h of treatment, the model group and positive control group were given LPS (Sigma Inc., Shenzhen, China), and the final concentrations were adjusted to 0.5 µg/mL. After 48 h, the results of PEG-2 and COX-2 were determined by ELISA kits (Nanjing Jiancheng Bioengineering Insitute, Nanjing, China).

#### 3.4.3. Testing the Anti-Insulin Resistance Activities of **1**–**3** against a Mouse Insulinoma Cell Line

For the anti-insulin resistance activity assay, INS-1 cells were trypsinized and seeded onto 96-well plates at a density of 1 × 10^4^ cells per well. Then, we chose cells whose survival rate was between 80–90% and the cells were treated with the target compounds (50 μM for compound **1**, 5 μM for compound **2**, 25 μM for compound **3**) in 100 μL of culture medium containing 40 mmol/L glucose in sextuplicates, and metformin was used (Sigma Inc., Shenzhen, China) as a positive control. After 48 h of treatment, the supernatant was removed, and 100 μL of sugar-free culture medium was seeded and settled for 30 min. Then, each group was seeded in 100 μL of culture medium containing 16.7 mM glucose and incubated for 1 h at 37 °C. The results were determined by Rat-Insulin ELISA kits (Nanjing Jiancheng Bioengineering Insitute, Nanjing, China).

## 4. Conclusions

In summary, we have synthesized three novel target products, **1**–**3**, which combined two pharmacophores from berberine, magnolol, and metformin. All the products were synthesized and their anti-inflammatory activities and antidiabetic activities against INS-1 cells and RAW264.1 cells were evaluated in vitro for the first time. It is noteworthy that the crystal structures of the target product **2** and the intermediate product **7b** were reported for the first time, and this method provided an inexpensive, safe, and simple way to synthesize 1,3,5-triazine derivatives. Compounds **1**–**3** all showed remarkable anti-inflammatory and antidiabetic effects in INS-1 cells and RAW264.1 cells compared with ibuprofen and metformin. The results demonstrated that compounds **1**–**3** were able to relieve the COX-2/PEG-2 destruction and stimulate an insulin secretion increment. Summarizing the obtained results in our study, we believe that compounds **1**–**3** have great potential as anti-inflammatory and antidiabetic agents. Moreover, various pharmacophoric groups were well-tolerated under the optimized reaction conditions; thus, they may serve as drug leads in pharmaceutical chemistry.

## Supplementary Materials

The following are available online.
