# Supplementary material for: Synthesis, Characterization, and Biological Evaluations of 1,3,5-Triazine Derivatives of Metformin Cyclization with Berberine and Magnolol in the Presence of Sodium Methylate
Source: Molecules. 2017 Oct 18;22(10):1752. doi: 10.3390/molecules22101752 (PMC6151379; doi:10.3390/molecules22101752)

Supporting Information for

Synthesis, characterization and biological evaluations of 1,3,5- triazine derivatives of metformin cyclization with berberine and magnolol in the presence of sodium methylate

Han Cao,† Shili Liao,† Wenjing Zhong,† Xuerong Xiao,† Jiancheng Zhu,† Weimin Li*,‡ Xia Wu*,† Yifan Feng*,†

†School of Pharmacy, Guangdong Pharmaceutical University, Guangzhou 510000, P. R. China

‡ School of Guangzhou University of Chinses Medicine, Guangzhou 510000, P. R. China.

Author Contributions: Han Cao and Wenjing Zhong designed the research; Han Cao, Wenjing Zhong and Shili Liao performed experiments; Han Cao and Xia Wu analyzed the data, Han Cao, Xuerong Xiao, Jiancheng Zhu and Weimin Li wrote or contributed to the writing of the manuscript.

* Correspondence to E-mail: 13925023915@139.com; wxiaxia@163.com; yffeng@139.com

Contents

1. Cellular proliferation assay 4

2. Physical/chemical properties of compounds **1-3** and **7** 12

^{1}H NMR assignments of compounds 1-3 14

^{13}CNMR assignments of compounds 1-3 15

Figure S1. ^{1}H NMR (500 MHz, DMSO-*d6*) spectrum of 1 17

Figure S2.^{13}C NMR (500 MHz, DMSO-*d6*) spectrum of 1 18

Figure S3. HSQC spectrum of 1 in DMSO-*d6* 19

Figure S4. HSBC spectrum of 1 in DMSO-*d6* 20

Figure S5. ^{1}H-^{1}H COSY spectrum of 1 in DMSO-*d6* 21

Figure S6. HR-ESI-MS spectrum of 1 22

Figure S7. IR spectrum of 1 23

Figure S8. UV spectrum of 1 24

Figure S9.^{1}H NMR (500 MHz, DMSO-*d6*) spectrum of 2 25

Figure S10. ^{13}C NMR (500 MHz, DMSO-*d6*) spectrum of 2 26

Figure S11. HSQC spectrum of 2 in DMSO-*d6* 27

Figure S12. HMBC spectrum of 2 in DMSO-*d6* 28

Figure S13. ^{1}H-^{1}H COSY spectrum of 2 in DMSO-*d6* 29

Figure S14.HR-ESI-MS spectrum of 2 30

Figure S15. IR spectrum of 2 31

Figure S16.UV spectrum of 2 32

Figure S17. ^{1}H NMR (500 MHz, DCOOD-*d2*) spectrum of 3 33

Figure S18.^{13}C NMR (500 MHz, DCCOD-*d2*) spectrum of 3 34

Figure S19.HSQC spectrum of 3 in DCCOD-*d2* 35

Figure S20. HMBC spectrum of 3 in DCCOD-d_2....36
Figure S21. ^{1}H-^{1}H COSY spectrum of 3 in DCCOD-d_2....37
Figure S22.HR-ESI-MS spectrum of 3....38
Figure S23. IR spectrum of 3....39
Figure S24. UV spectrum of 3....40
Figure S25. ^{1}H NMR (500 MHz, DMSO-d_6) spectrum of 7b....41
Figure S26.^{13}C NMR (500 MHz, DMSO-d_6) spectrum of 7b....42
Figure S27.HSQC spectrum of 7b in DMSO-d_6....43
Figure S28. HMBC spectrum of 7b in DMSO-d_6....44
Figure S29. ^{1}H-^{1}H COSY spectrum of 3 in DMSO-d_6....45
Figure S30. ^{1}H NMR (500 MHz, DMSO-d_6) spectrum of 10....46
Figure S31.HSQC spectrum of 10 in DMSO-d_6....48
Figure S32. HMBC spectrum of 10 in DMSO-d_6....49
Figure S33. ^{1}H-^{1}H COSY spectrum of 10 in DMSO-d_6....50
Figure S34. ^{1}H NMR (500 MHz, DMSO-d_6) spectrum of 9....51
Figure S35.^{13}C NMR (500 MHz, DMSO-d_6) spectrum of 9....52
Figure S36. ^{1}H-^{1}H COSY spectrum of 9 in DMSO-d_6....53
Figure S37.HSQC spectrum of 9 in DMSO-d_6....54
Figure S38. HMBC spectrum of 9 in DMSO-d_6....55

1. Cellular proliferation assay

Table S1. Cytotoxicities of compounds **1-3** against RAW264.7 cells

Compound	5μM (%)	10μM (%)	25μM (%)	50μM (%)	100μM (%)	200μM (%)	400μM (%)	800μM (%)	1600μM (%)
1	131.51±1.30	100.23±10.60	84.59±2.47	81.97±1.20	75.17±4.20	66.34±9.87	33.27±8.57	22.46±8.91	13.85±12.00
2	101.38±10.00	104.56±10.20	109.87±6.00	92.74±12.00	86.84±10.00	85.17±14.10	32.49±21.20	10.45±19.60	11.80±10.00
3	74.27±+7.11	61.16±10.48	58.07±23.40	51.53±23.00	39.16±11.00	36.50±13.90	33.55±32.70	28.12±14.70	24.99±11.00

Table S2. Cytotoxicities of compounds **1-3** against INS-1 cells

Compound	5μM (%)	10μM (%)	25μM (%)	50μM (%)	100μM (%)	200μM (%)	400μM (%)	800μM (%)	1600μM (%)
1	73.71±12.81	63.16±+3.00	61.11±5.60	60.73±2.00	57.26±5.80	55.47±4.80	54.16±+2.00	48.59±4.30	37.44±11.00
2	90.51±3.68	89.99±5.70	87.22±5.90	87.09±8.90	86.12±8.80	80.50±6.00	55.88±10.00	25.60±15.00	26.96±12.00
3	72.64±4.53	70.13±7.80	61.44±3.40	59.08±3.10	56.89±4.30	53.62±6.50	52.81±2.90	51.98±4.50	46.09±3.70

Table S3. Anti-inflammatory effects of compounds **1-3**

Compound	PEG-2	COX-2
Blank	34.7±4.35**	0.576±0.01**
Model	65.94±3.43	0.850±0.05
Ibuprofen	38.04±3.50**	0.574±0.03**
1	48.10±5.67**	0.739±0.04**
2	44.67±4.38**	0.729±0.07**
3	41.93±3.38**	0.634±0.09**

**Compared model group, p <0.01

Table S4. Antidiabetic effects of compounds **1-3**

Compound	Insulin content (ng/mL)
Blank	0.24±0.01*
Metformin	0.23±0.02*
Model	0.16±0.01
1	0.23±0.03*

2	0.25±0.02*
3	0.22±0.02*

*Compared model group, $p < 0.05$

Table S5. Optimization of the reaction conditions[a] for compounds **7**

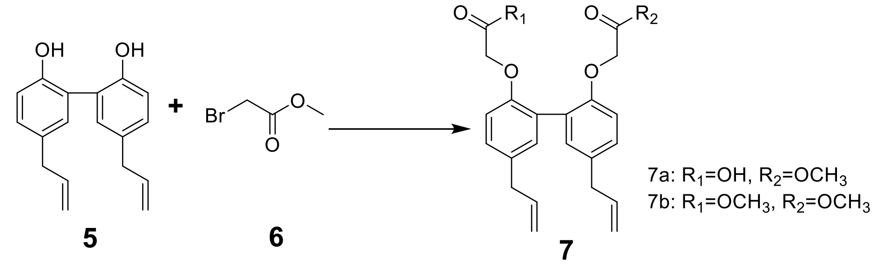

Entry	Base	Solvent	Temp (°C)	Time (h)	Yield (%) (7a + 7b)
1	Na_2CO_3	MeOH	25	6	57
2	Na_2CO_3	MeOH	45	6	64
3	Na_2CO_3	MeOH	65	6	75
4	Na_2CO_3	MeOH	75	6	70
5	K_2CO_3	MeOH	65	8	55
6	NaOH	MeOH	65	8	31
7	Na_2CO_3	CH_3CN	65	8	33
8	Na_2CO_3	DMF	65	8	nr[b]
9	Na_2CO_3	MeOH	65	2	56

10	Na_2CO_3	MeOH	65	4	63
11	Na_2CO_3	MeOH	65	6	75
12	Na_2CO_3	MeOH	65	8	59

[a] Unless otherwise specified, the reactions to produce compounds **7** were performed with **5** (0.01 mol), **6** (2 mL, 0.02 mol) and base (0.02 mol) in 50 mL of solvent.
[b] nr: The reaction did not occur.

Table S6. Optimization of the reaction conditions[c] for compound **10**

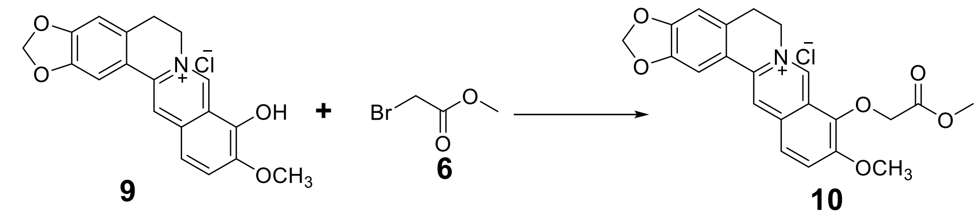

Entry	Solvent	Temp (°C)	Time (h)	Yield[d] (%)
1	DCM	25	6	49
2	DCM	45	6	78

3	DCM	55	6	72
4	CH_3CN	45	6	64
5	DMF	45	6	41
6	DCM	45	2	69
7	DCM	45	4	77
8	DCM	45	6	78
9	DCM	45	8	80
10	DCM	45	10	68

[b] nr: The reaction did not occur.

[c] Unless otherwise specified, the reactions to produce compounds **10** were performed with **9** (0.01 mol) and **6** (4 mL, 0.04 mol) in 20 mL of solvent.

[d] Yield of the isolated product.

Table S7. Optimization of the reaction conditions[e], [f] of compounds **1-3**

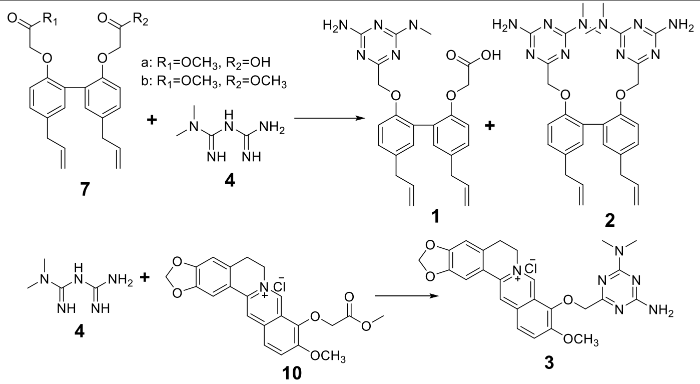

Entry	Base	Solvent	Temp (°C)	Time (h)	Yield [e],[f] (%)(1+2)	Yield (%)(3)
1	CH_3ONa	MeOH	25	8	20	nr [b]
2	CH_3ONa	MeOH	45	8	21	8
3	CH_3ONa	MeOH	65	8	23	27
4	CH_3ONa	MeOH	75	8	22	23
5	Na_2CO_3	MeOH	65	10	nr [b]	nr [b]
6	K_2CO_3	MeOH	65	10	nr [b]	nr [b]
7	CH_3ONa	CH_3CN	65	10	22	nr [b]

8	CH_3ONa	DMF	65	10	19	nr [b]
9	CH_3ONa	MeOH	65	2	20	23
10	CH_3ONa	MeOH	65	6	21	26
11	CH_3ONa	MeOH	65	10	25	28
12	CH_3ONa	MeOH	65	12	38	33
13	CH_3ONa	MeOH	65	16	37	27

[b] nr: The reaction did not occur.

[c] Unless otherwise specified, the reactions to produce compounds **1** and **2** were performed with **4** (0.04 mol), **7** (0.01 mol), and base (0.04 mol) in 70 mL of solvent.

[d] Unless otherwise specified, the reaction of compounds **3** were performed with **4** (0.025mol), **10** (0.01 mol), and base (0.04 mol) in 75 mL of solvent.

[e] Yield of the isolated products.

[f] Total yields of products **1** and **2**.

2. Physical/chemical properties of compounds **1-3** and **7**

Compound 1: Colorless needles; mp 277-280 °C; UV: (MeOH) λmax (log ε): 219, 281 nm; IR (νmax): 3225.36, 3076.87, 2929.34, 1680.66, 1630.52, 1571.7, 1402.96, 1275.68, 1216.86, 1133.94, 1056.8, 991.23, 912.165, 808.028 cm^{-1}; HRESIMS m/z 476.2322 M^+, calculated 476.2298.

Compound 2: White solid; mp 211-213 °C; UV: (MeOH) λmax (log ε): 217, 279 nm; IR (νmax): 3478.95, 3298.64, 3148.22, 2923.56, 1639.2, 1571.7, 1524.45, 1497.45, 1402.96,1216.86, 1136.83, 1068.37, 997.98, 912.165, 808.02 cm^{-1}; HRESIMS m/z 569.3101 M^+, calculated 569.3101.

Compound 3: Red solid; mp 215-217 °C; UV: (MeOH) λmax (log ε): 229, 284, 324 nm; IR (νmax): 1674.87, 1600.63, 1533.38, 1491.67, 1405.85, 1237.11, 1038.48, 935.306, 772.351 cm^{-1}; HRESIMS m/z 473.1936 M^+, calculated 473.1937.

Crystal data for **compound 1:** $C_{26}H_{25}N_5O_4$, *M* = 471.51, triclinic, *a* = 10.8613(3) Å, *b* = 11.5697(3) Å, *c* = 11.9513(3) Å, α = 103.651°, β = 115.085°, γ = 94.003°, *V* = 1296.89(7) Å^3, *T* = 100(10)K, space group P-1, *Z* = 2, μ(CuKα) = 0.684 mm^{-1}, 26893 reflections collected, independent reflections (R_{int} = 0.0612). The final R_1 value was 0.0628 ($I > 2\sigma(I)$). The final $wR(F^2)$ value was 0.1742 ($I > 2\sigma(I)$). The final R_1 value was 0.0657 (all data). The final $wR(F^2)$ was 0.1777 (all data). The goodness of fit on F^2 was 1.070. The CCDC number is 1547785.

Crystal data for **compound 7**: $C_{24}H_{26}O_6$, *M* = 410.66, triclinic, *a* = 7.8593(2) Å, *b* = 10.9479(2) Å, *c* = 13.3607(3) Å, α = 107.548°, β = 101.872°, γ = 92.095°, *V* = 1066.65(4) Å^3, *T* = 100(10) K, space group P-1, *Z* = 2, μ(CuKα) = 0.750 mm^{-1}, 34725 reflections measured, independent reflections (R_{int} = 0.1120). The final

R_1 value was 0.0777 ($I > 2\sigma(I)$). The final $wR(F^2)$ value was 0.1998 ($I > 2\sigma(I)$). The final R_1 value was 0.0618 (all data). The final $wR(F^2)$ was 0.2023 (all data). The goodness of fit on F^2 was 1.025. The CCDC number is 1547784.

^{1}H NMR assignments of compounds 1-3

Table S8. ^{1}H NMR data (500 MHz) for **1-3** in DMSO-d_6 and DCOOD-d_2 (δ in ppm, J in Hz)

No	1	2	3
1	3.30 t (7.5)	3.28 d (6.7)	7.17 s
1’	3.30 t (7.5)	3.28 d (6.7)	
2, 2’	5.88-5.99 m	5.90 ddt (16.8,10.0,6.8)	
3	4.98-5.04 m	5.057 s	5.84 s
3’	5.04-5.10 m	4.967 s	
5	6.90 d (8.4)	7.01, 7.02 dd (8.4,2.3)	2.17 s
5’	7.02-7.11 m	7.01, 7.02 dd (8.4,2.3)	
6	7.02-7.11 m	7.11 d (2.3)	3.83-3.87 m
8			6.41 s
8’	7.02-7.11 m	7.11 d (2.3)	
9	7.02-7.11 m	6.92 d (8.5)	
9’	6.86 d (8.4)	6.90 s	
10	4.69 s	4.71 s	
10’	4.55 s	4.71 s	
11			6.74 d (8.5)
12			6.94 d (8.5)
14		2.99 s	5.48 s
14’	2.99 s	2.99 s	
15		2.99 s	
15’	2.99 s	2.99 s	
16		6.80 s	
16’	6.80 s	6.80 s	
17			5.48 s
19			3.10 s
20			5.02 s
24			2.21 s

25		2.219 s
-NH_2		4.95 s

^{13}CNMR assignments of compounds 1-3

Table S9. ^{13}C NMR data (125 MHz) for 1-3 in DMSO-*d₆* and DCOOD-*d₂* (δ in ppm)

No	1	2	3
1	39.68	38.68	166.55
1'	39.68	38.68	
2	137.96	137.99	121.22
2'	137.96	137.99	
3	115.46	115.35	102.49
3'	115.46	115.35	
4	131.62	131.49	159.76
4'	131.52	131.49	
5	112.85	127.29	27.15
5'	131.37	127.29	
6	127.46	131.15	53.72
6'	127.13	131.15	
7	154.49	154.47	130.94
7'	153.80	154.47	
8	127.13	127.93	106.43
8'	127.46	127.93	
9	131.22	112.98	123.95
9'	112.56	112.98	
10	70.44	70.53	142.19
10'	65.64	70.53	
11		172.89	120.53
11'	170.56	172.89	
12	172.88	166.54	132.74
12'	166.54	166.54	
13		165.06	131.06
13'	165.07	165.06	
14		35.63	108.93

14’	35.64	35.63	
15		35.63	143.03
15’		35.63	
16			151.39
17			147.78
18			161.43
19			58.35
20			103.01
21			148.95
22			156.47
23			156.03
24			38.16
25			37.93

Figure S1. ^{1}H NMR (500 MHz, DMSO-d_6) spectrum of **1**

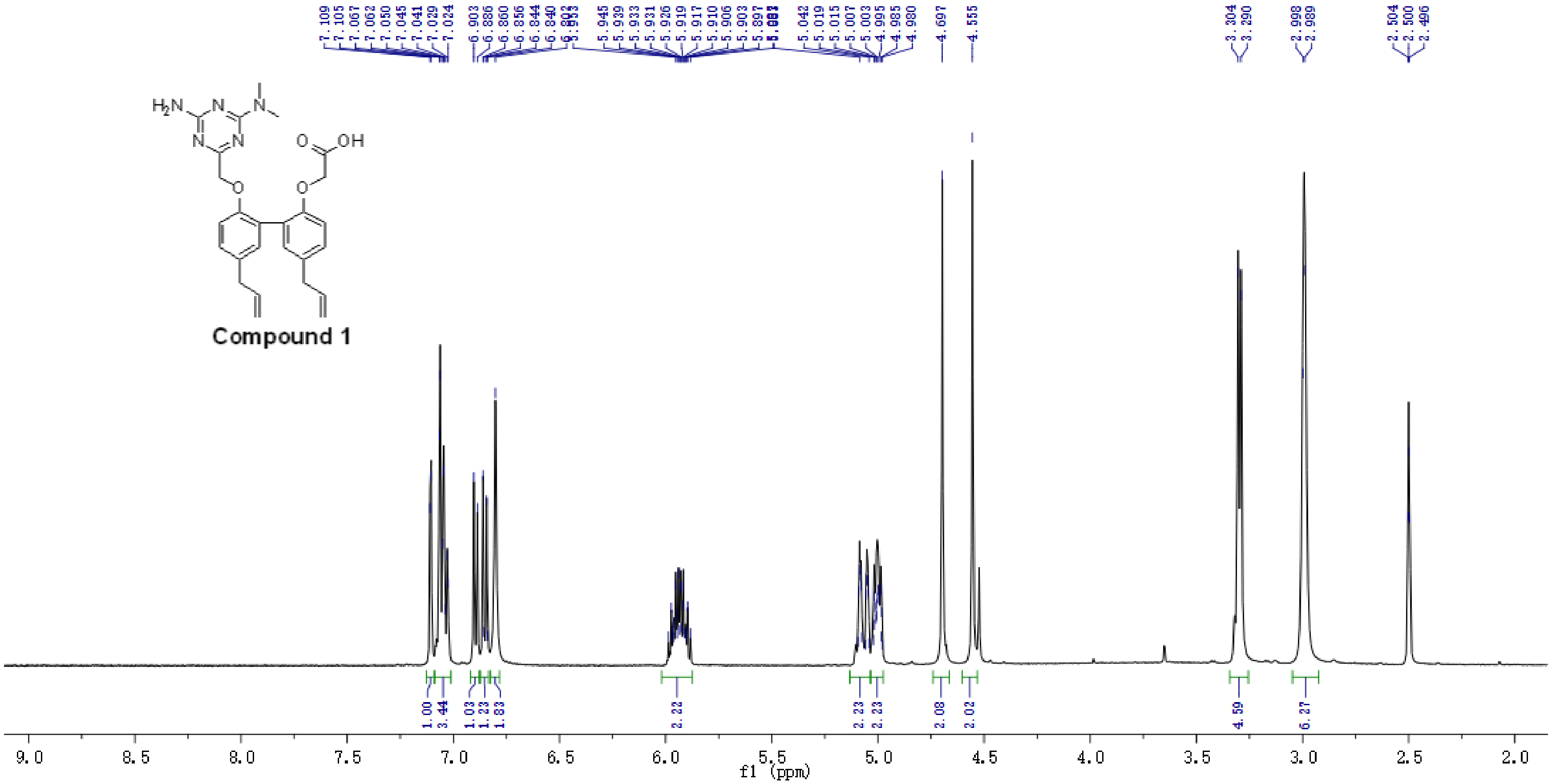

Figure S2.^{13}C NMR (125 MHz, DMSO-d_6) spectrum of **1**

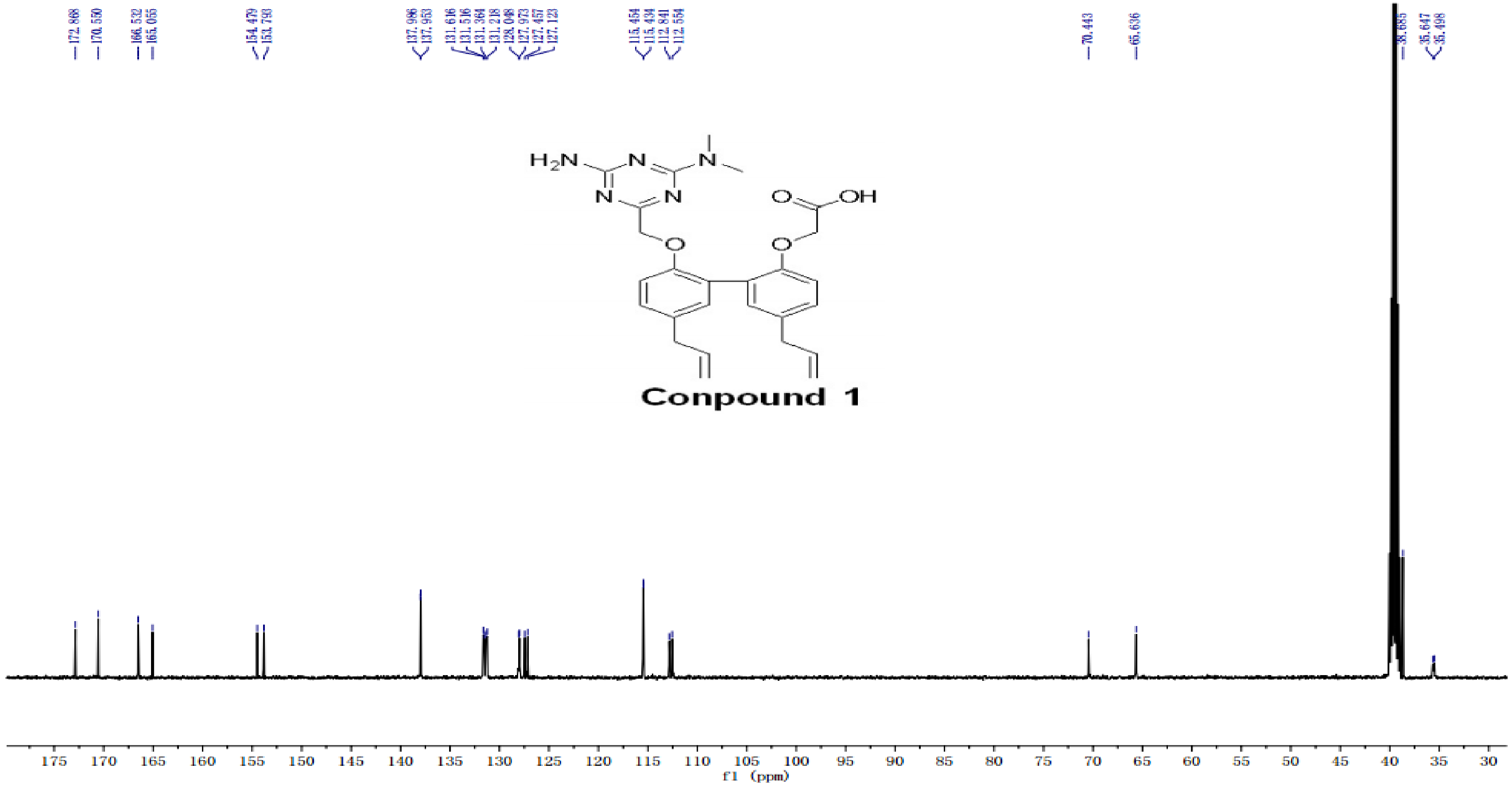

Conpound 1

Figure S3. HSQC spectrum of **1** in DMSO-d_6

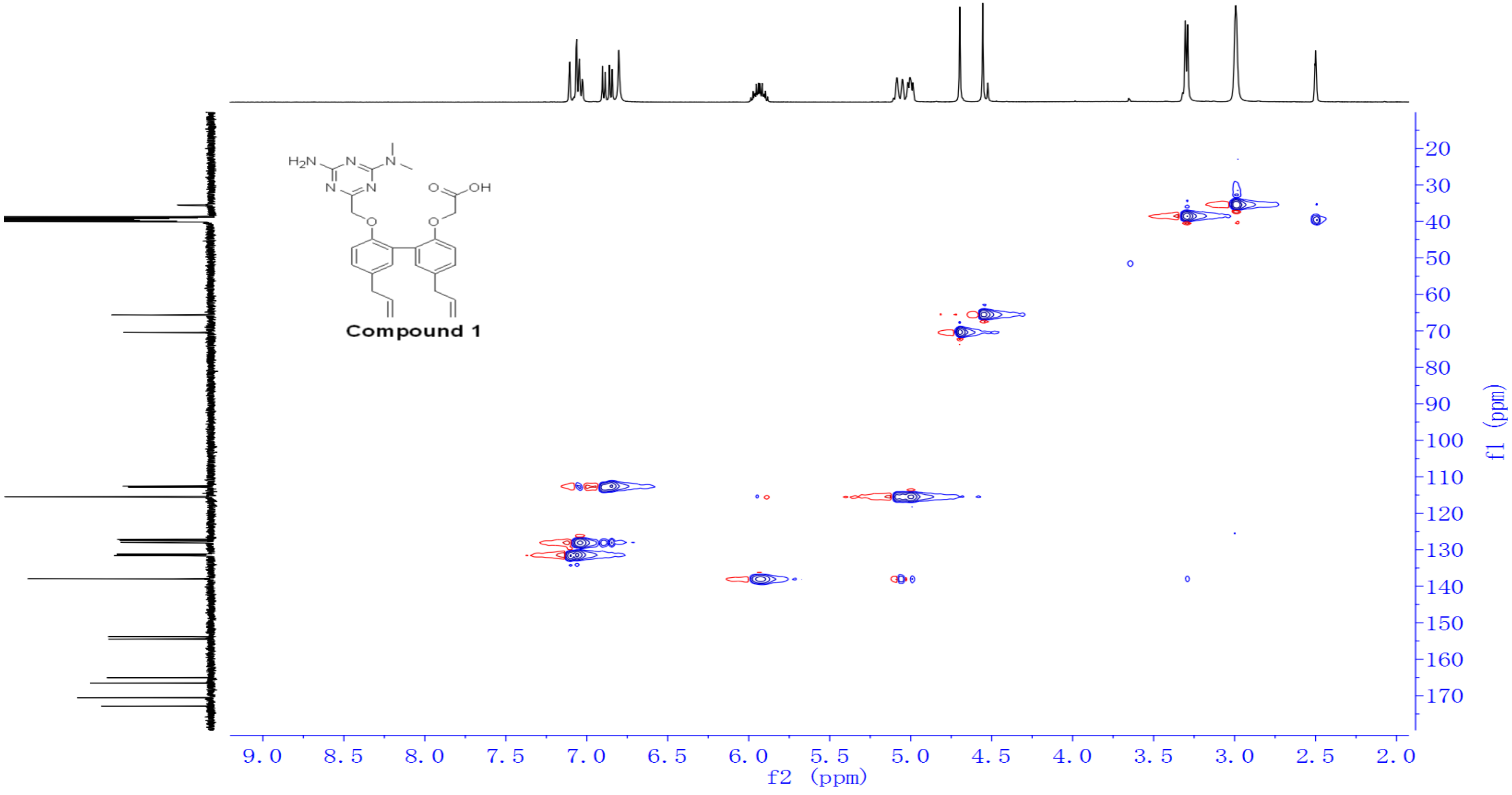

Figure S4. HMBC spectrum of **1** in DMSO-d_6

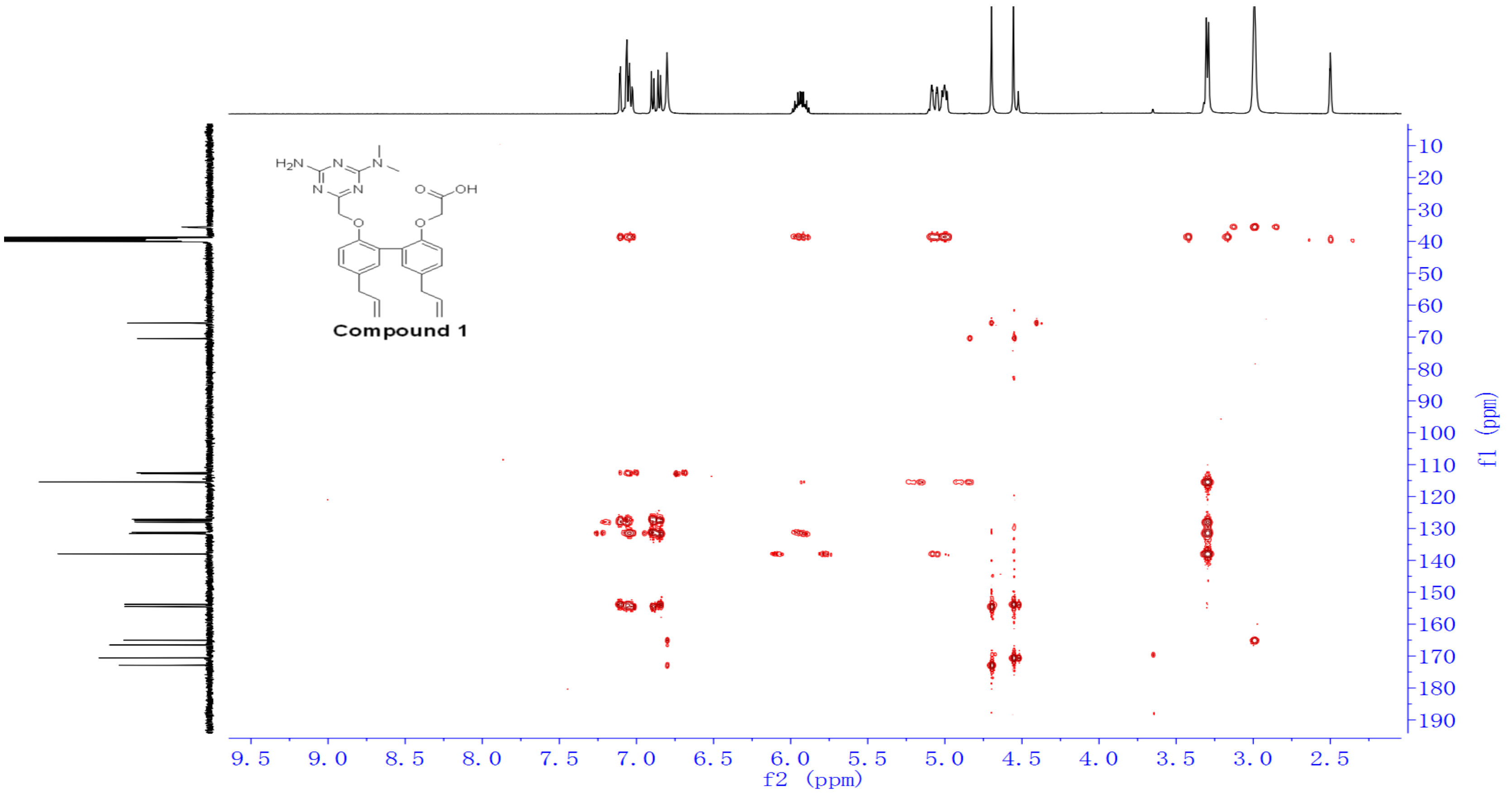

Figure S5. ^1H-^1H COSY spectrum of **1** in DMSO-*d*$_6$

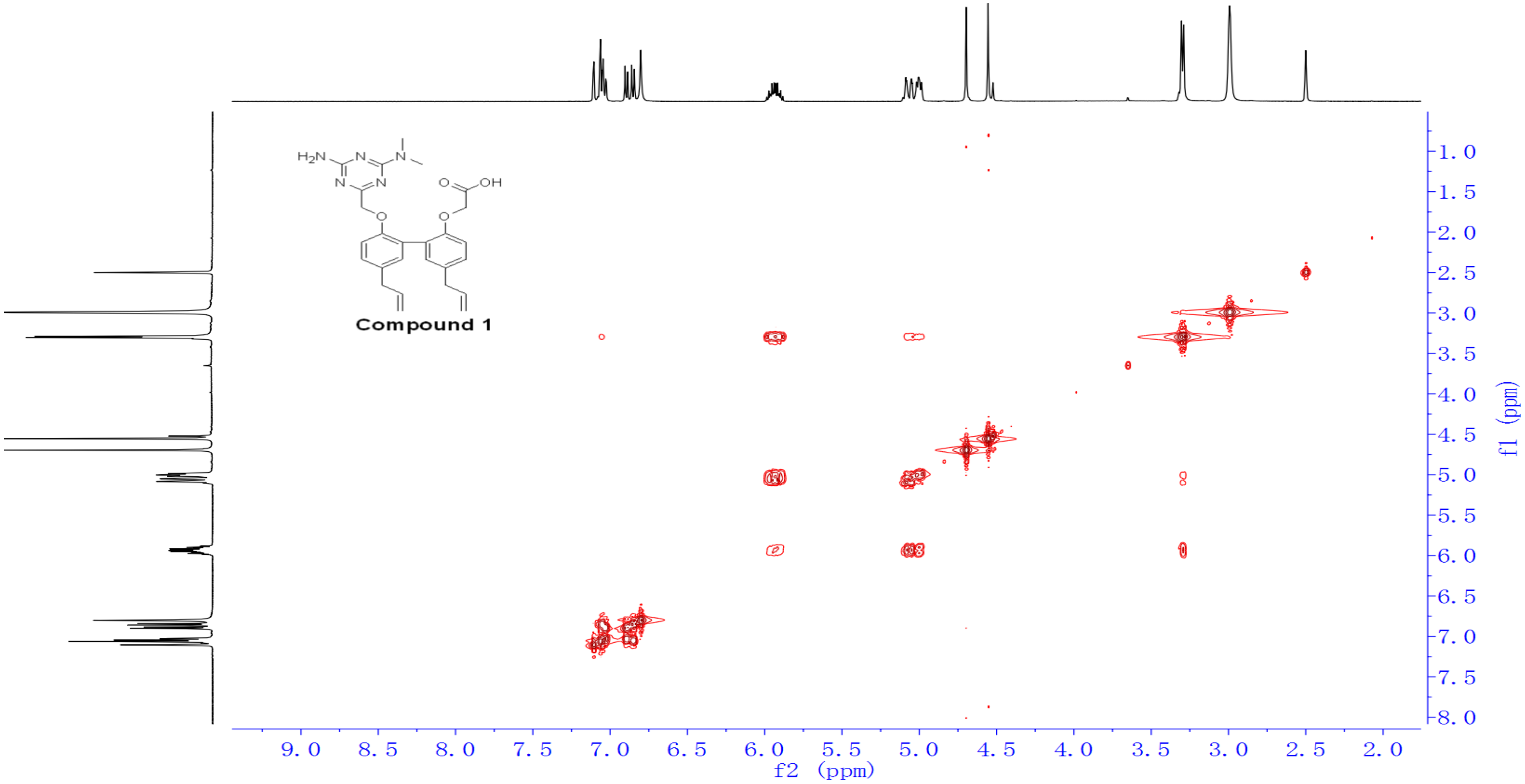

Figure S6. HR-ESI-MS spectrum of **1**

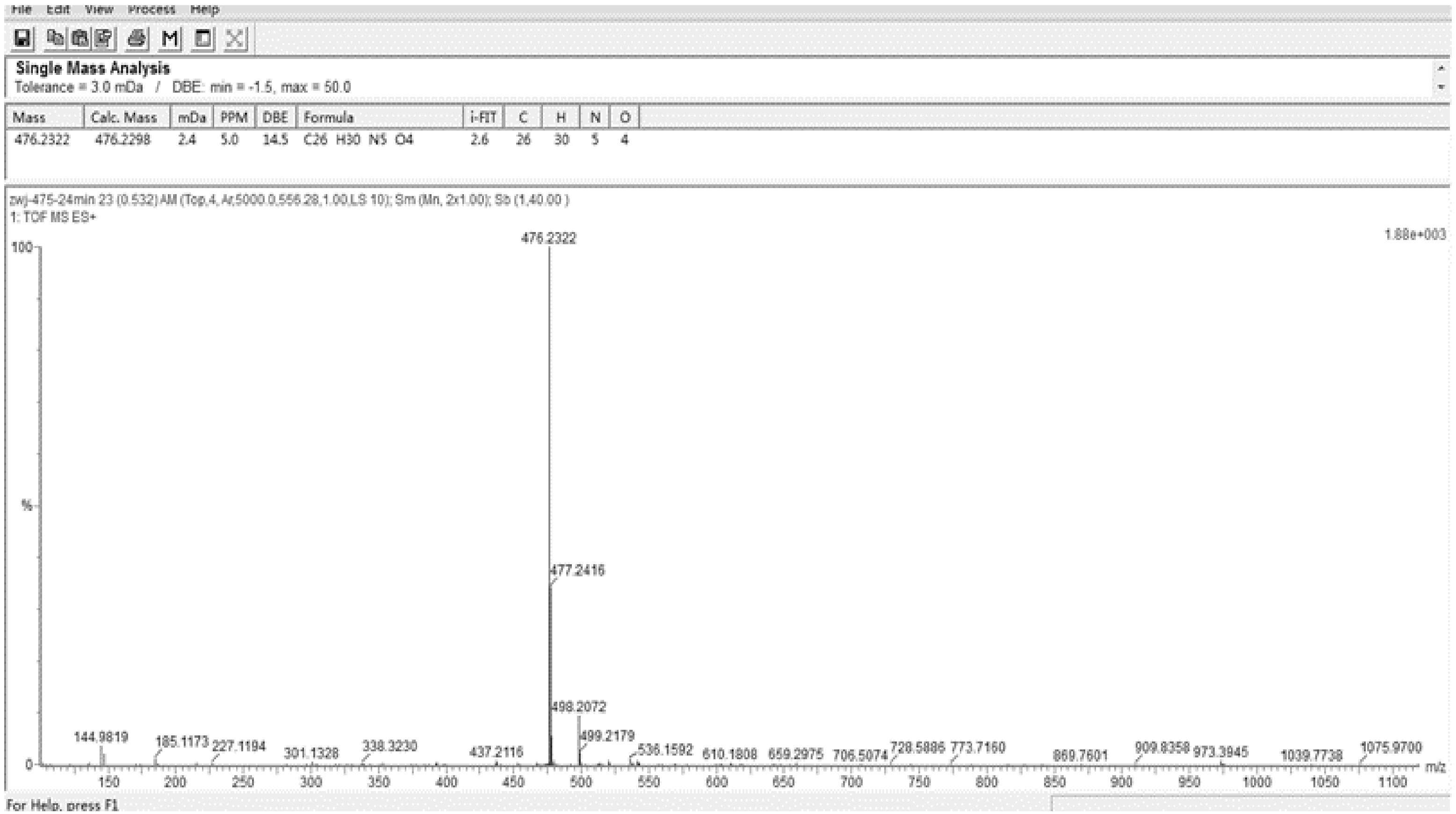

Figure S7. IR spectrum of **1**

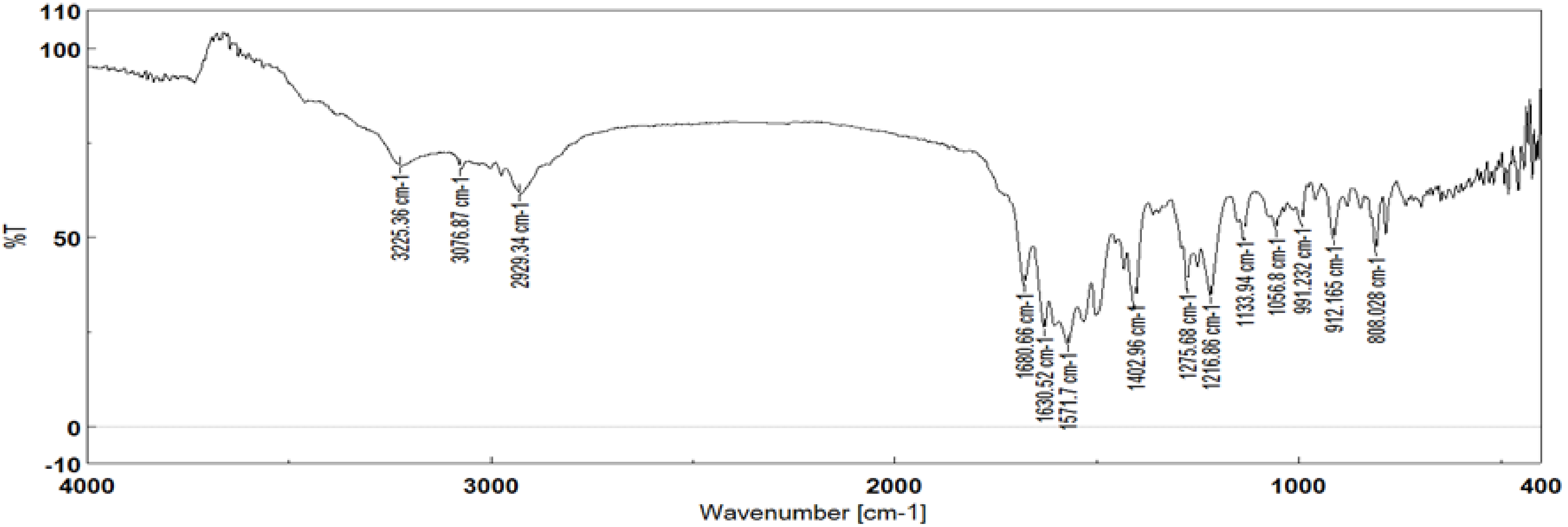

Figure S8. UV spectrum of **1**

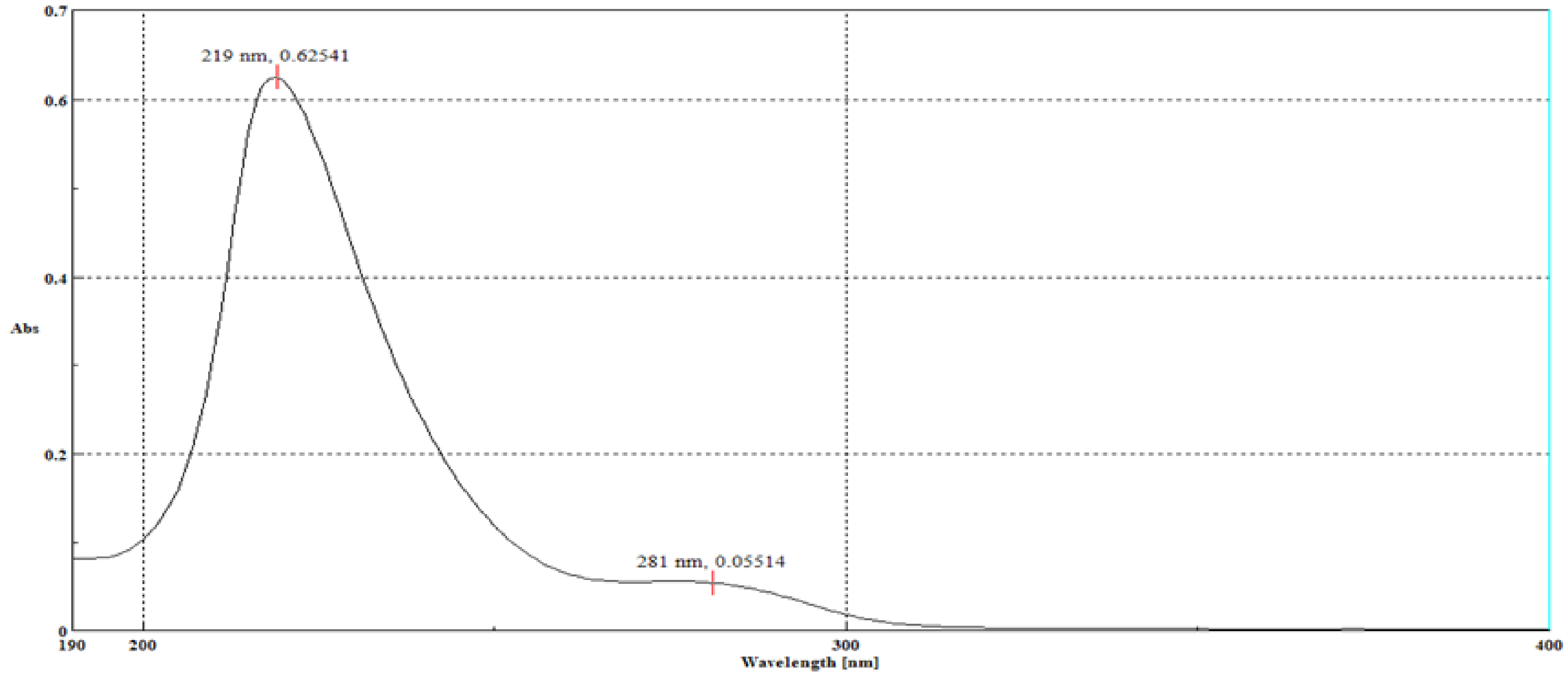

Figure S9. ^{1}H NMR (500 MHz, DMSO-d_6) spectrum of **2**

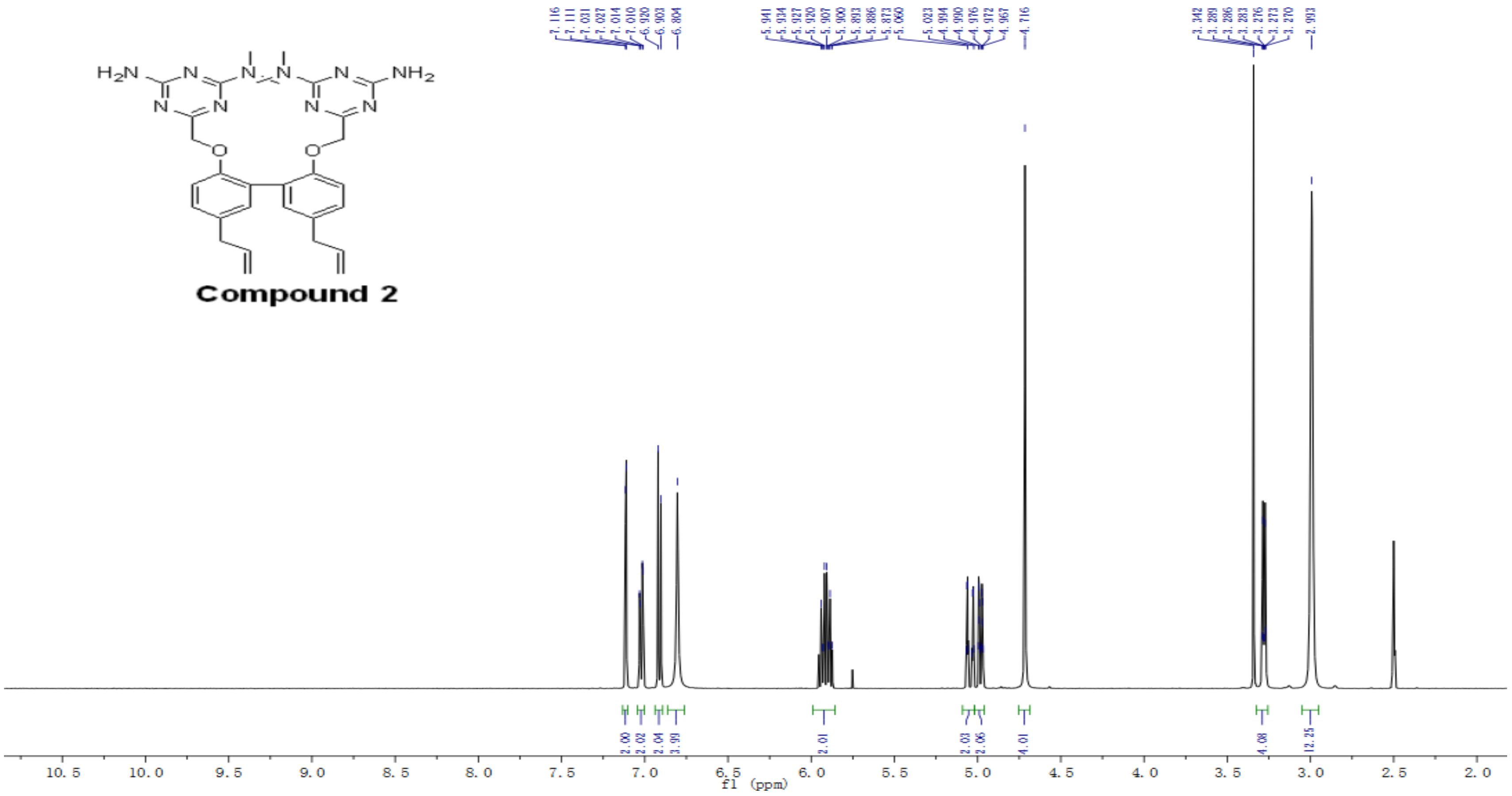

Figure S10. ^{13}C NMR (125 MHz, DMSO-d_6) spectrum of **2**

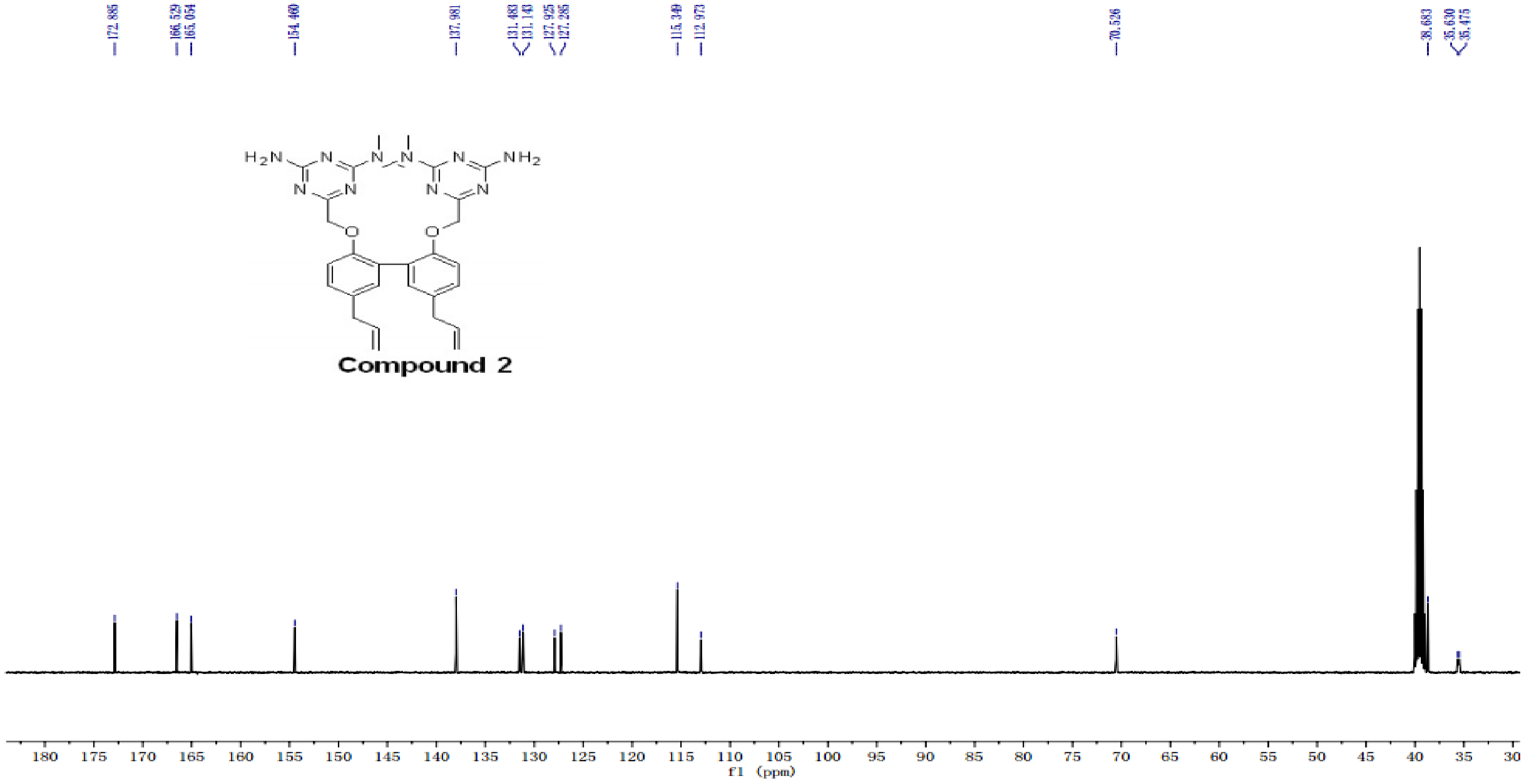

Figure S11. HSQC spectrum of **2** in DMSO-*d*$_6$

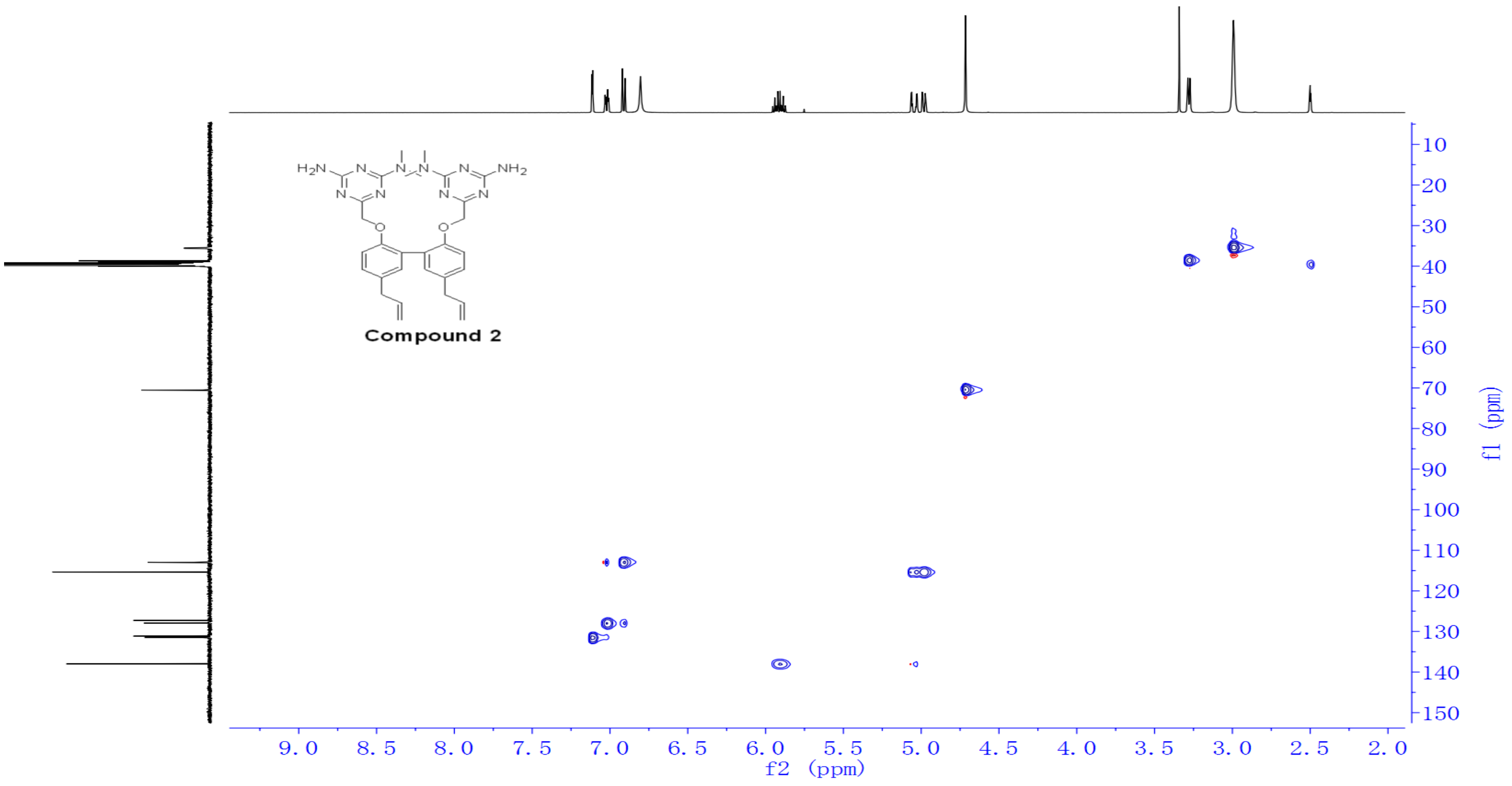

Figure S12. HMBC spectrum of **2** in DMSO-d_6

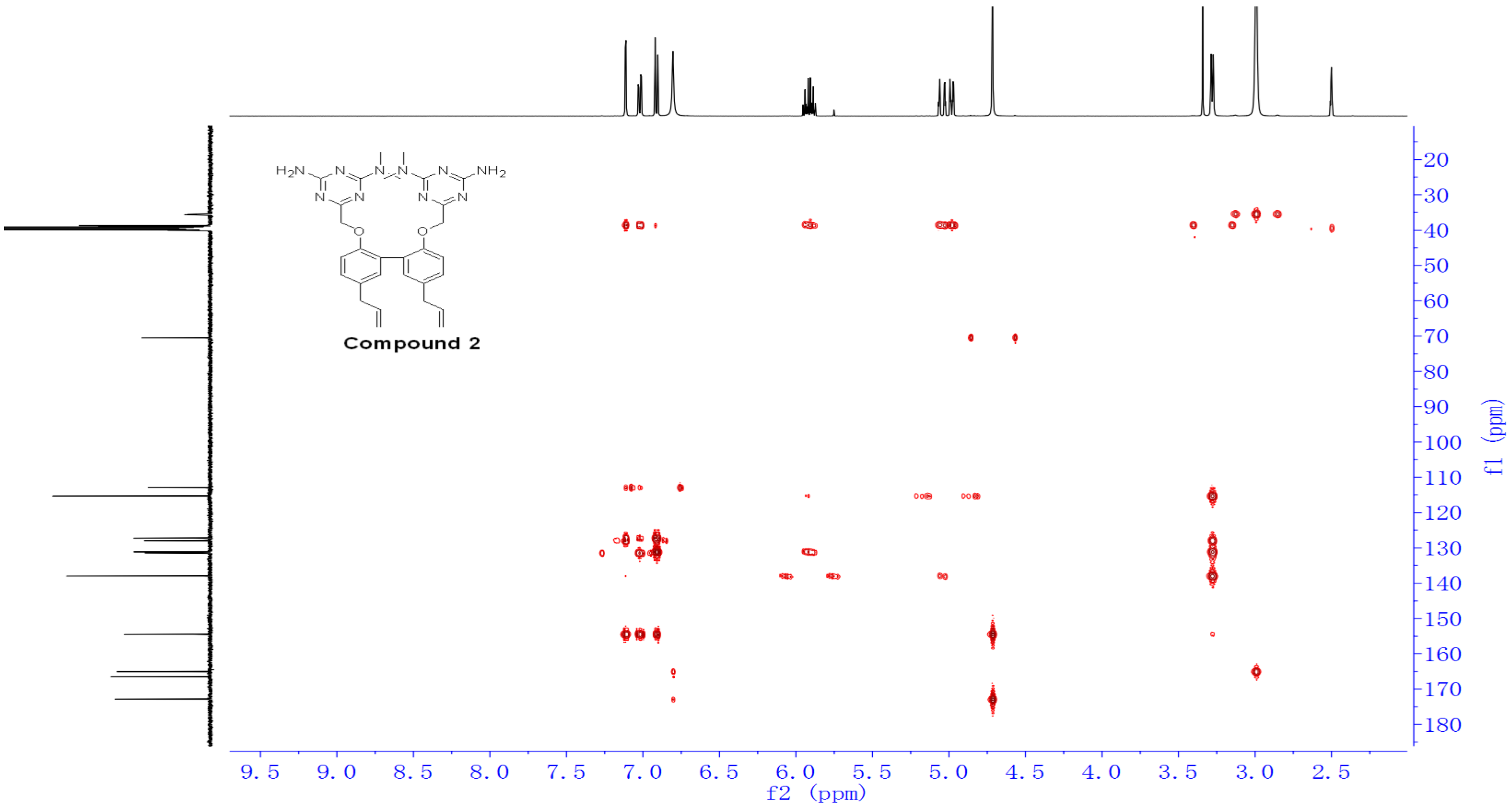

Figure S13. ^1H-^1H COSY spectrum of **2** in DMSO-d_6

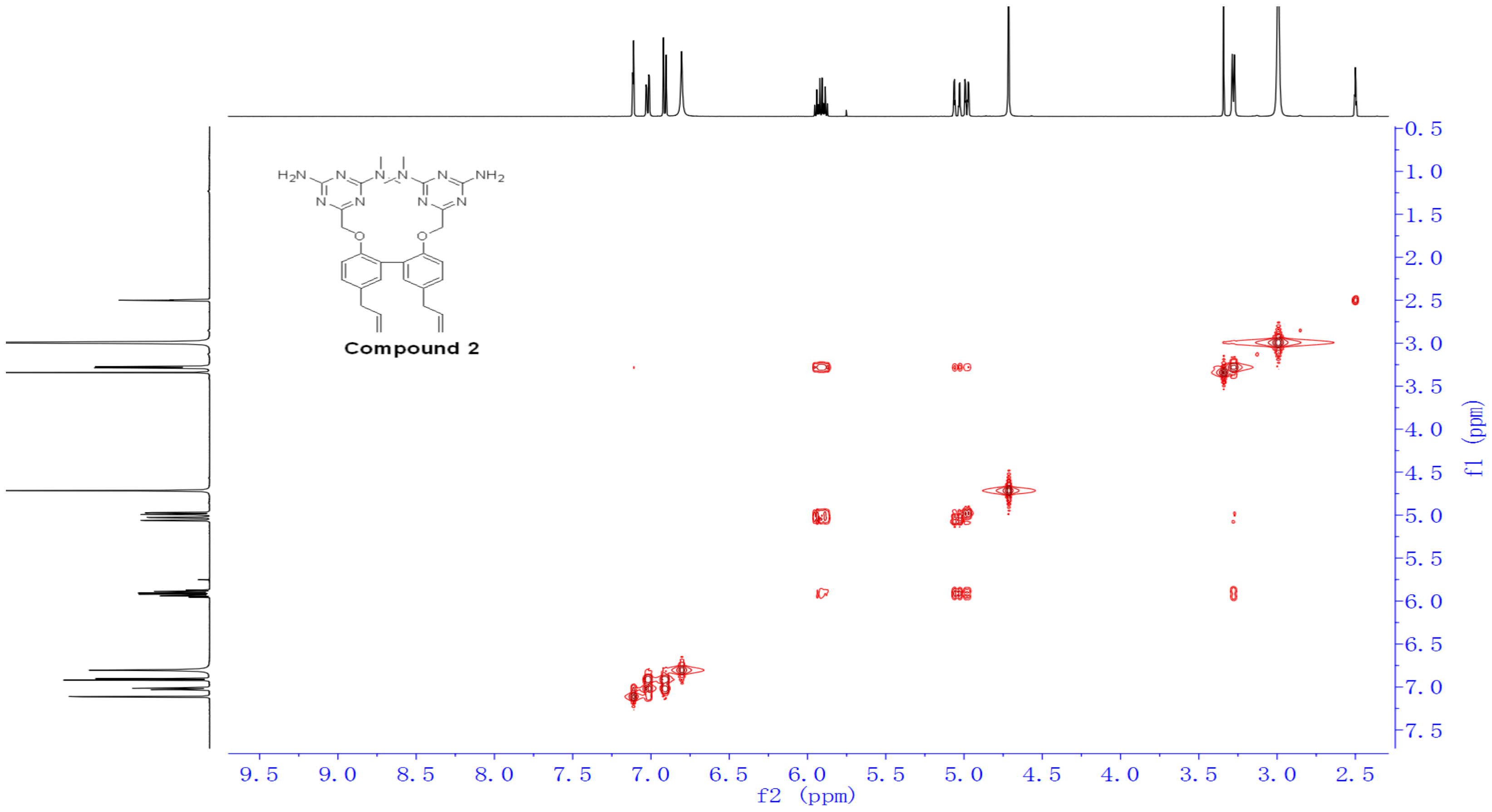

Figure S14.HR-ESI-MS spectrum of **2**

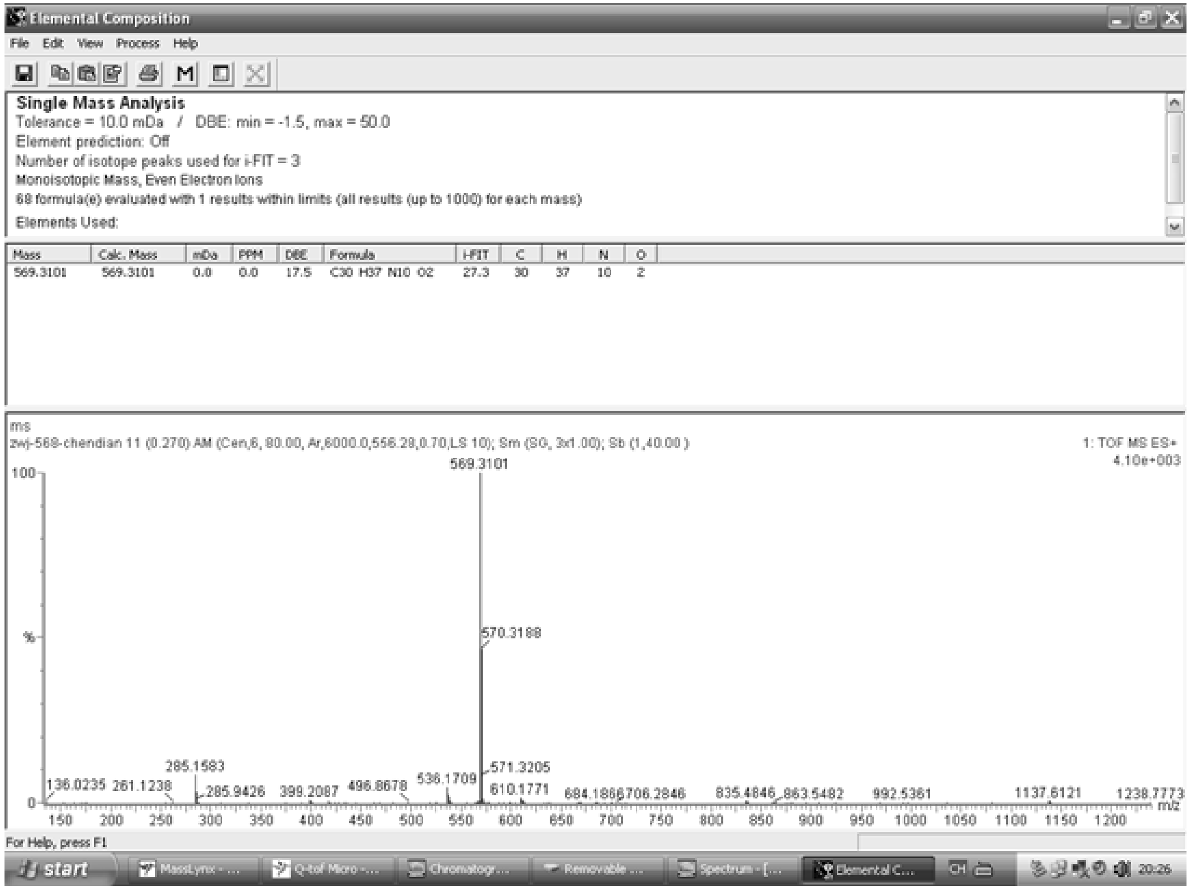

Figure S15. IR spectrum of **2**

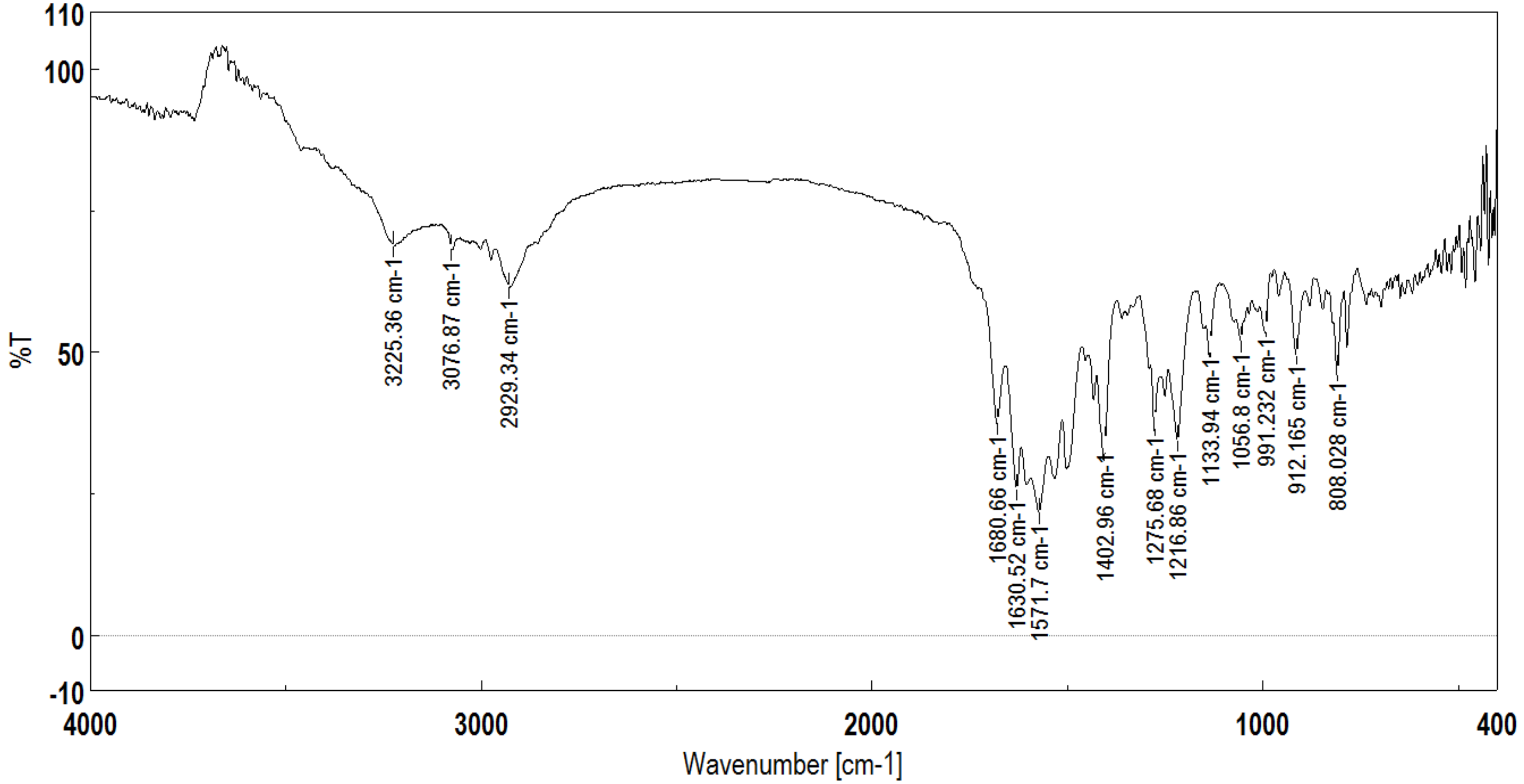

Figure S16.UV spectrum of **2**

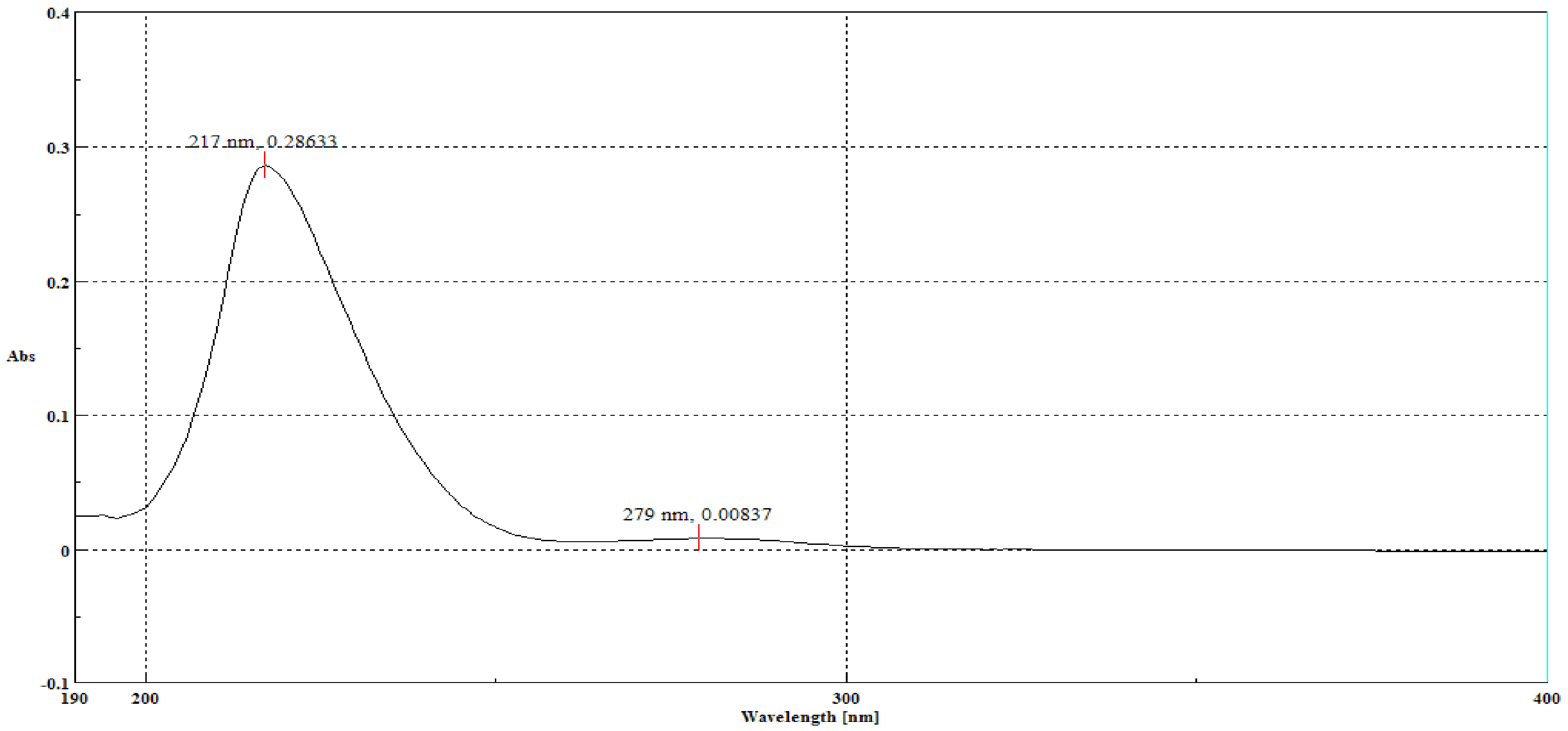

Figure S17. ^{1}H NMR (500 MHz, DCOOD-*d*$_2$) spectrum of **3**

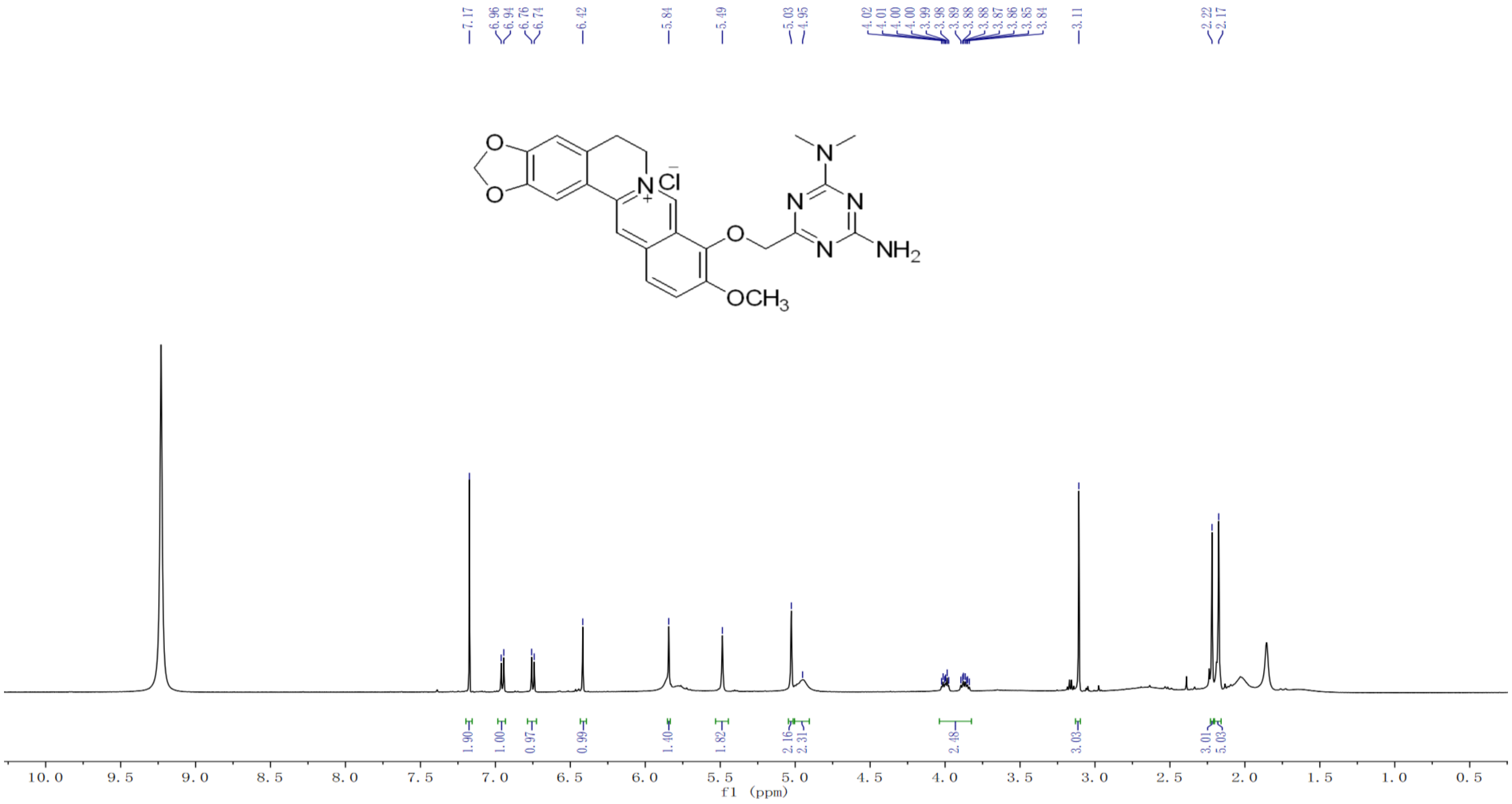

Figure S18. ^{13}C NMR (125 MHz, DCCOD-*d*$_2$) spectrum of **3**

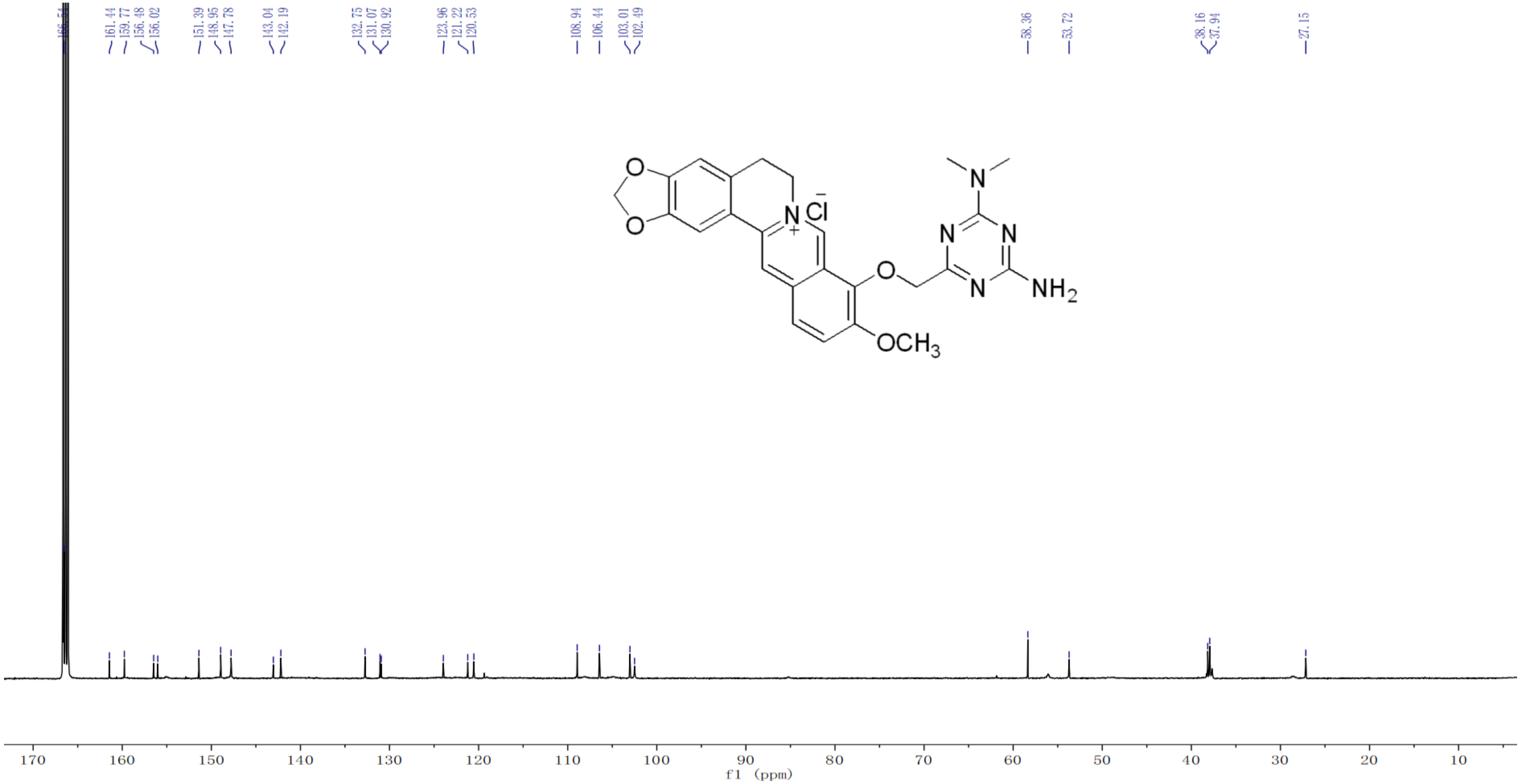

Figure S19. HSQC spectrum of **3** in DCCOD-d_2

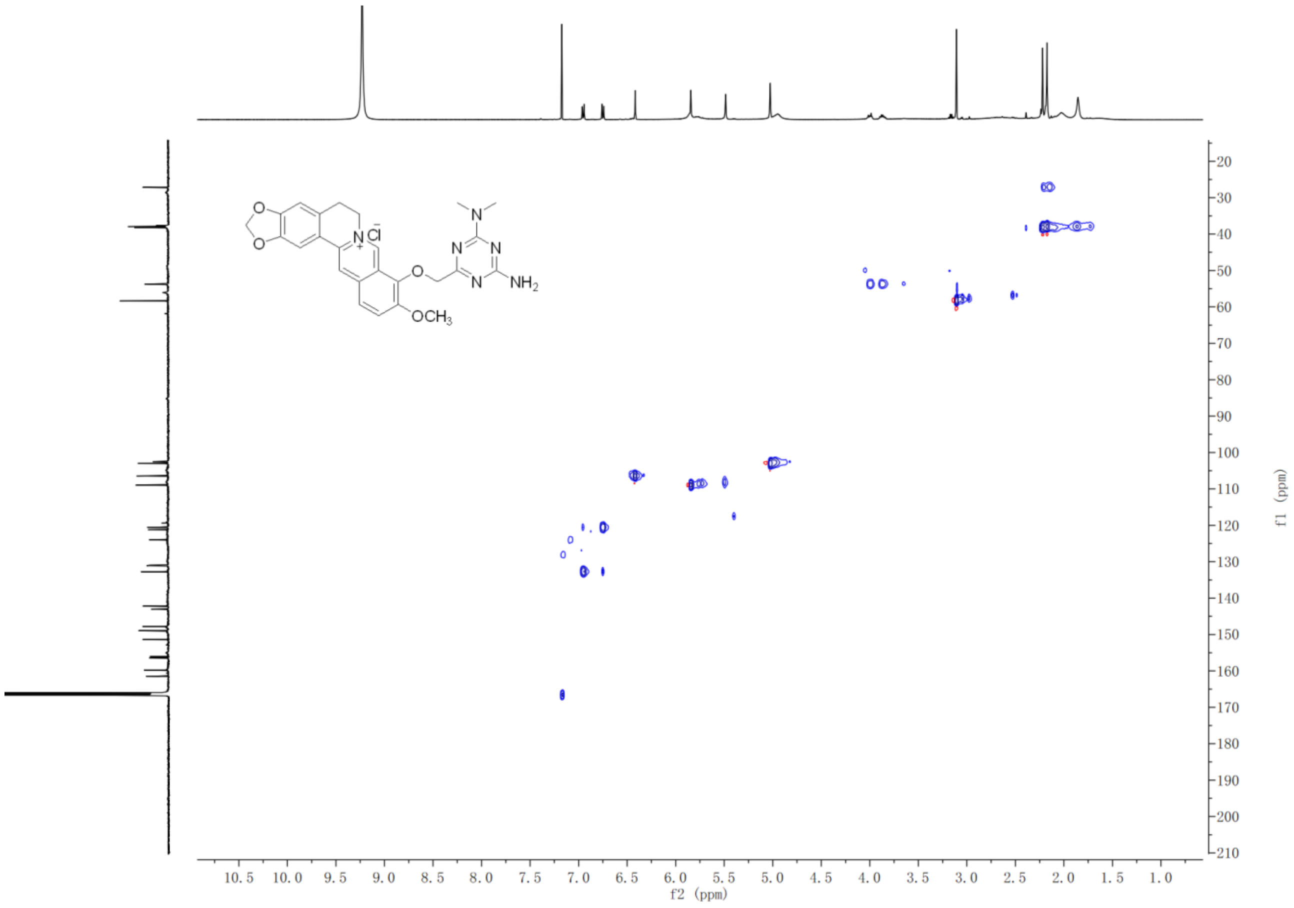

Figure S20. HMBC spectrum of **3** in DCCOD-d_2

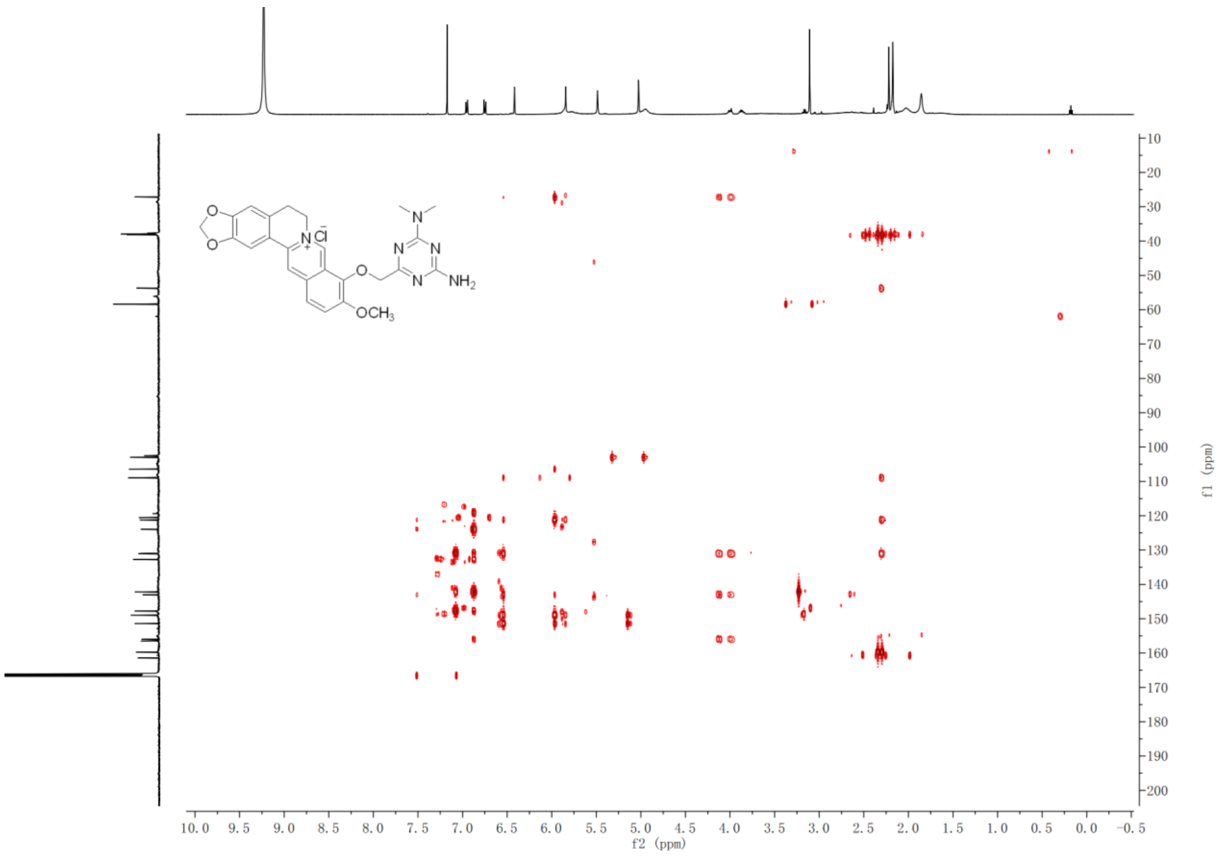

Figure S21. ^{1}H-^{1}H COSY spectrum of **3** in DCCOD-d_2

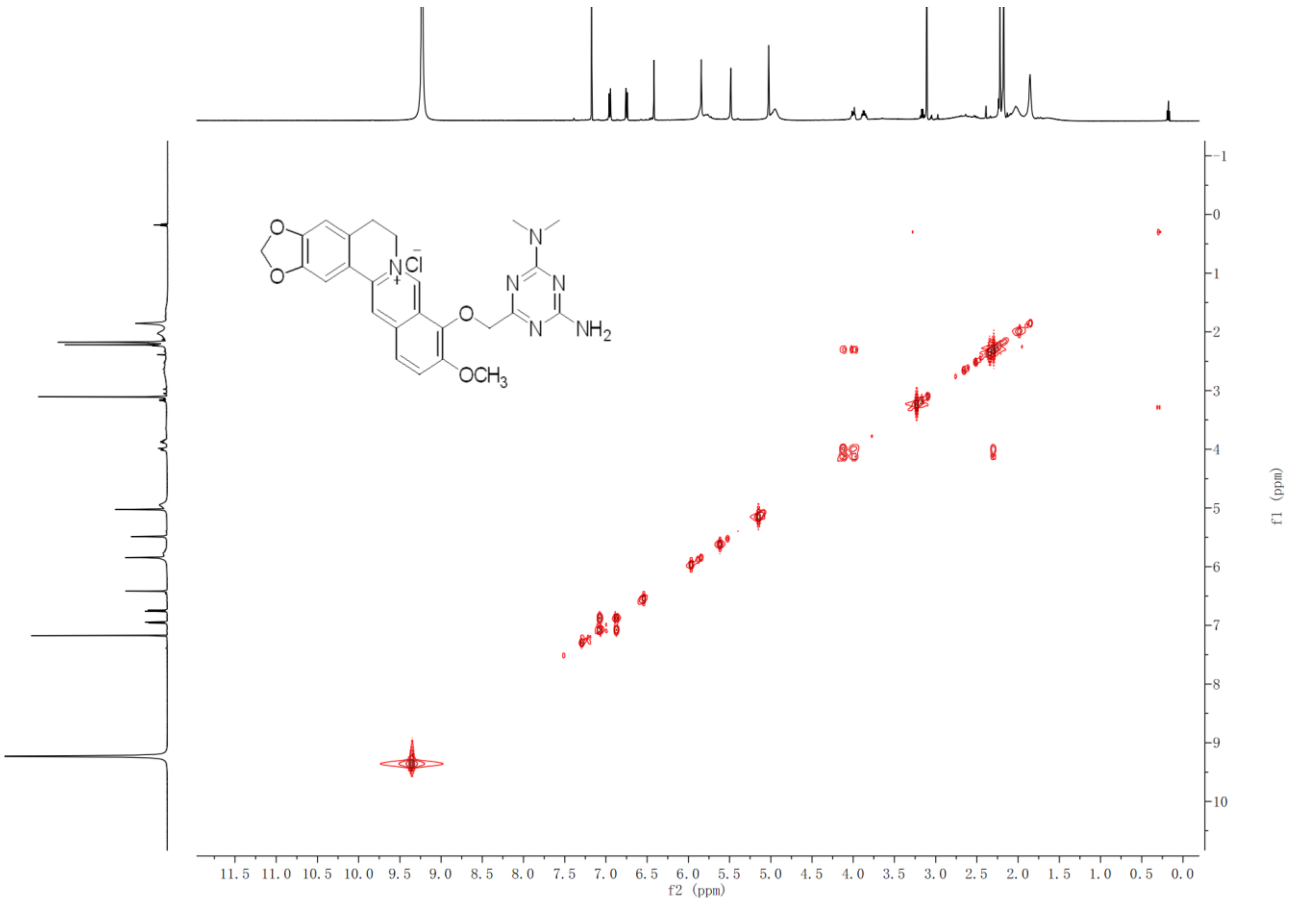

Figure S22. HR-ESI-MS spectrum of 3

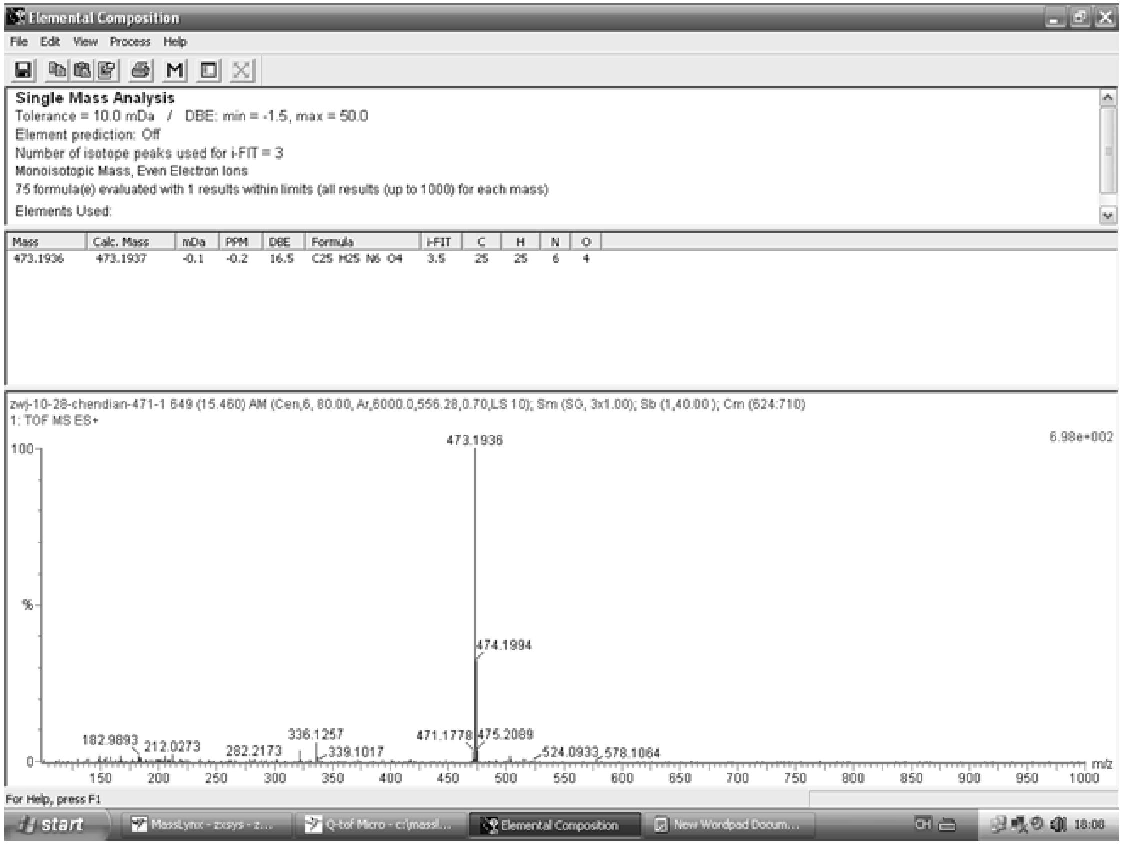

Figure S23. IR spectrum of **3**

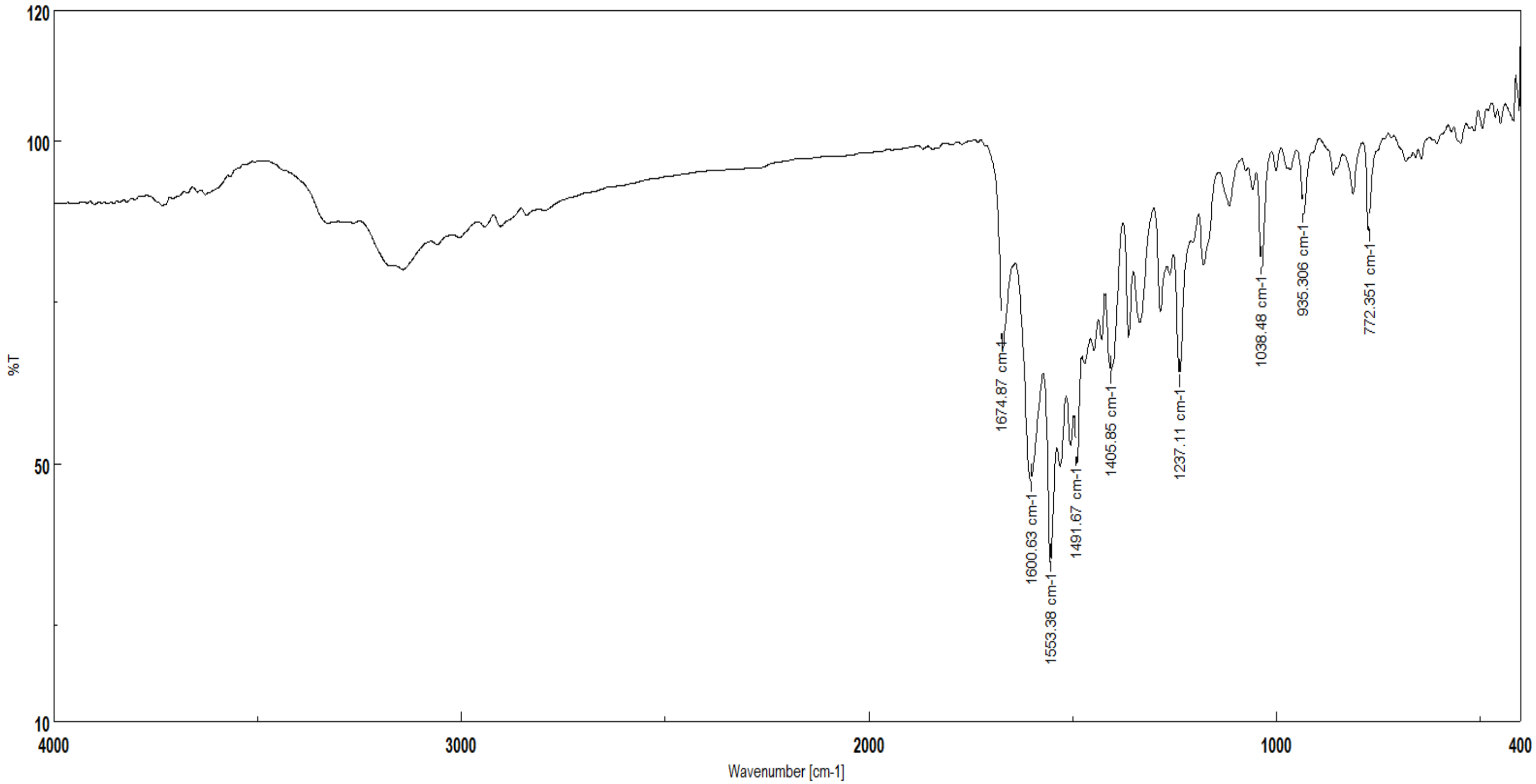

Figure S24. UV spectrum of **3**

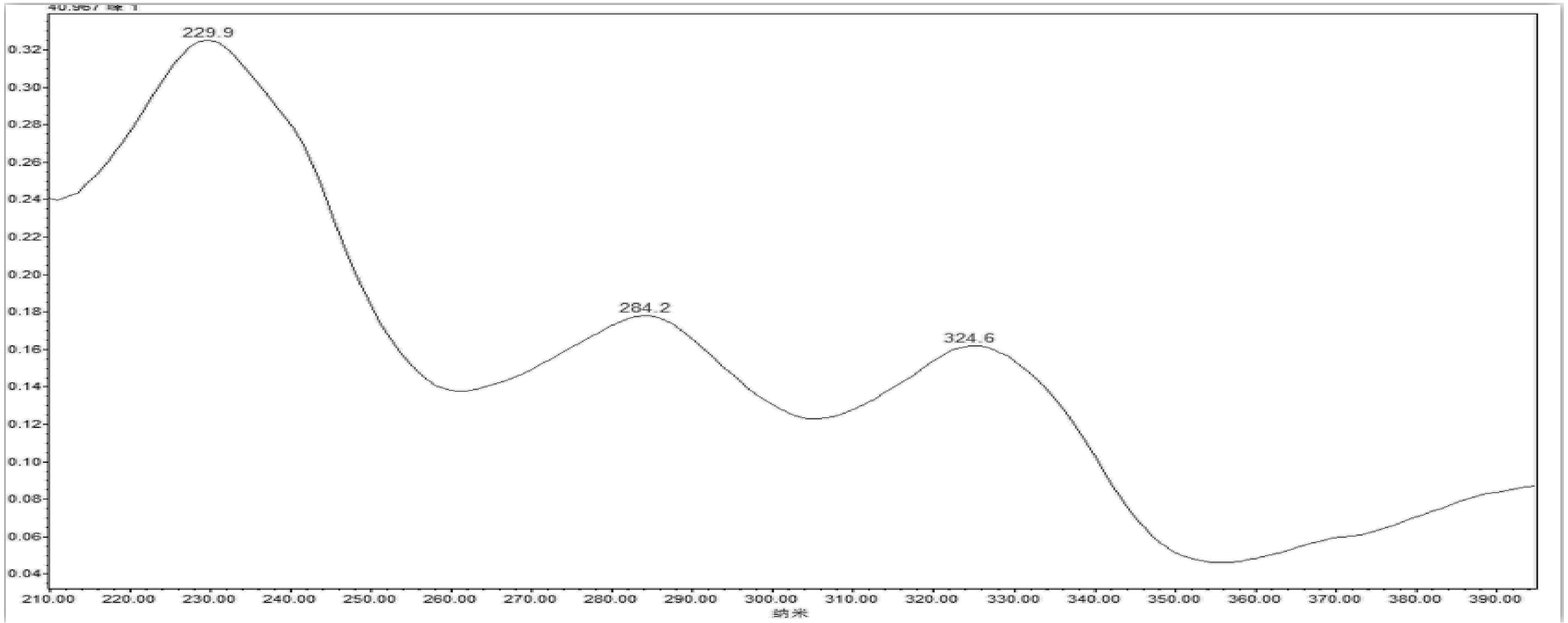

Figure S25. ^{1}H NMR (500 MHz, DMSO-d_6) spectrum of **7b**

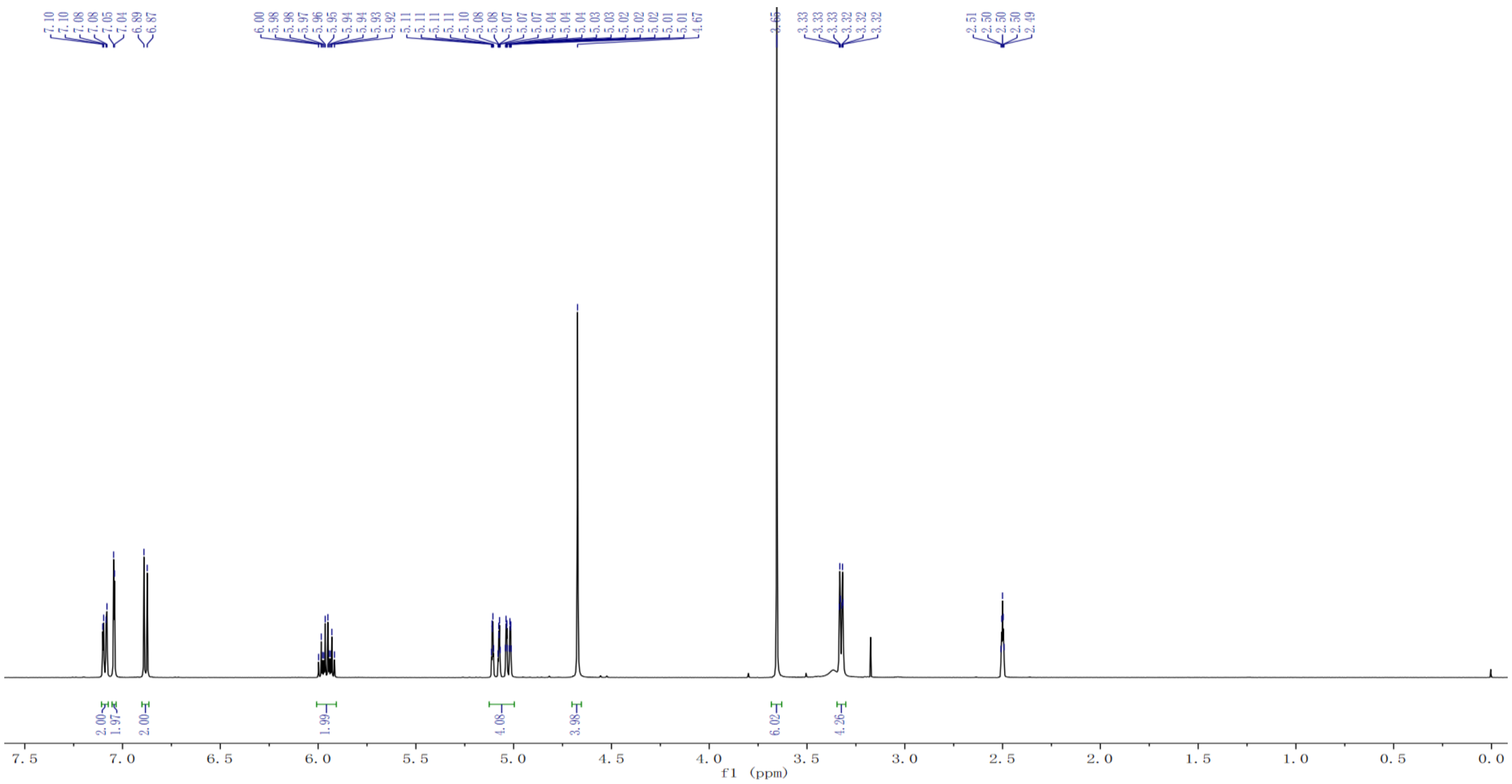

Figure S26.^{13}C NMR (125 MHz, DMSO-d_6) spectrum of **7b**

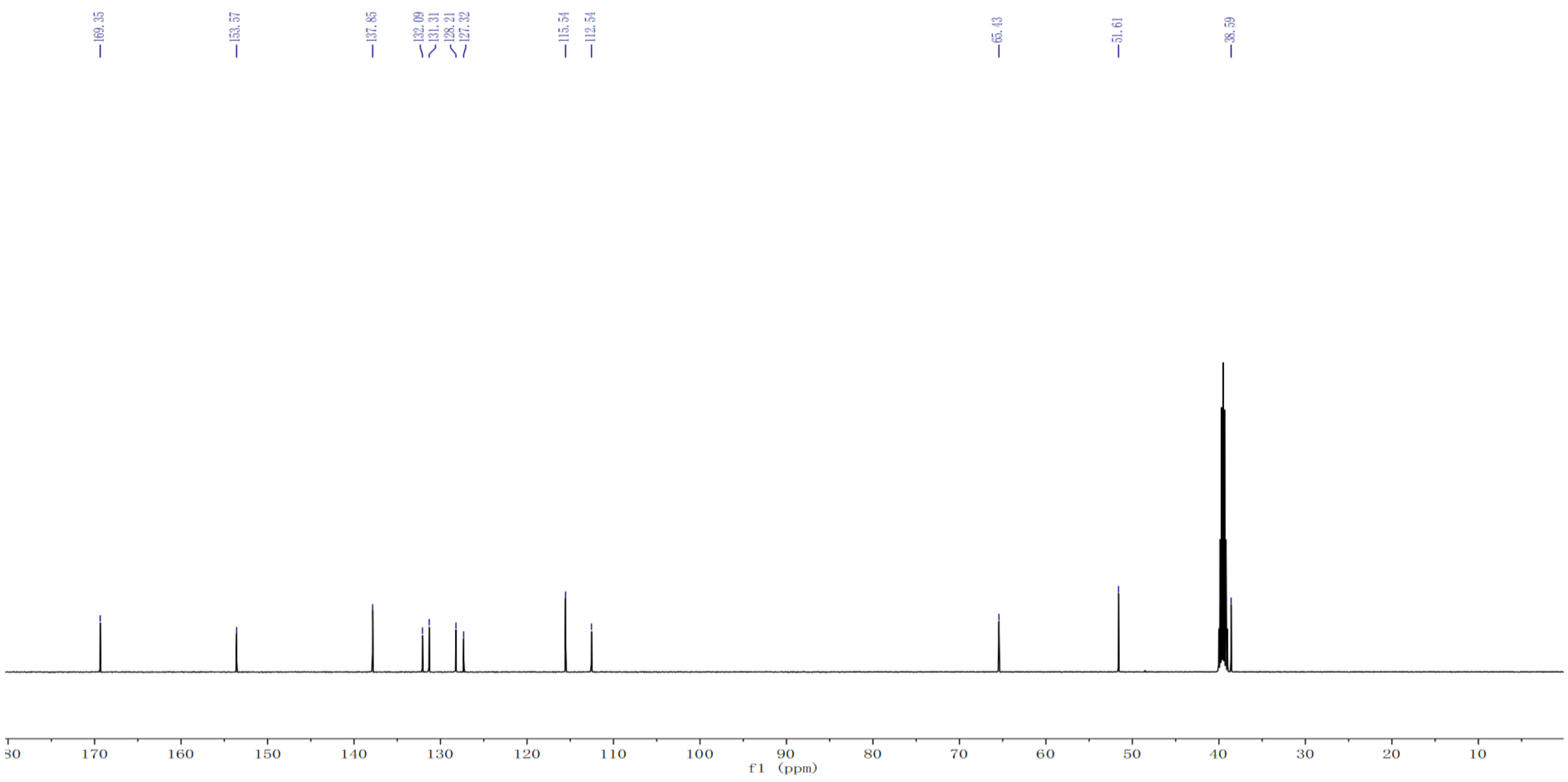

Figure S27.HSQC spectrum of **7b** in DMSO-*d*6

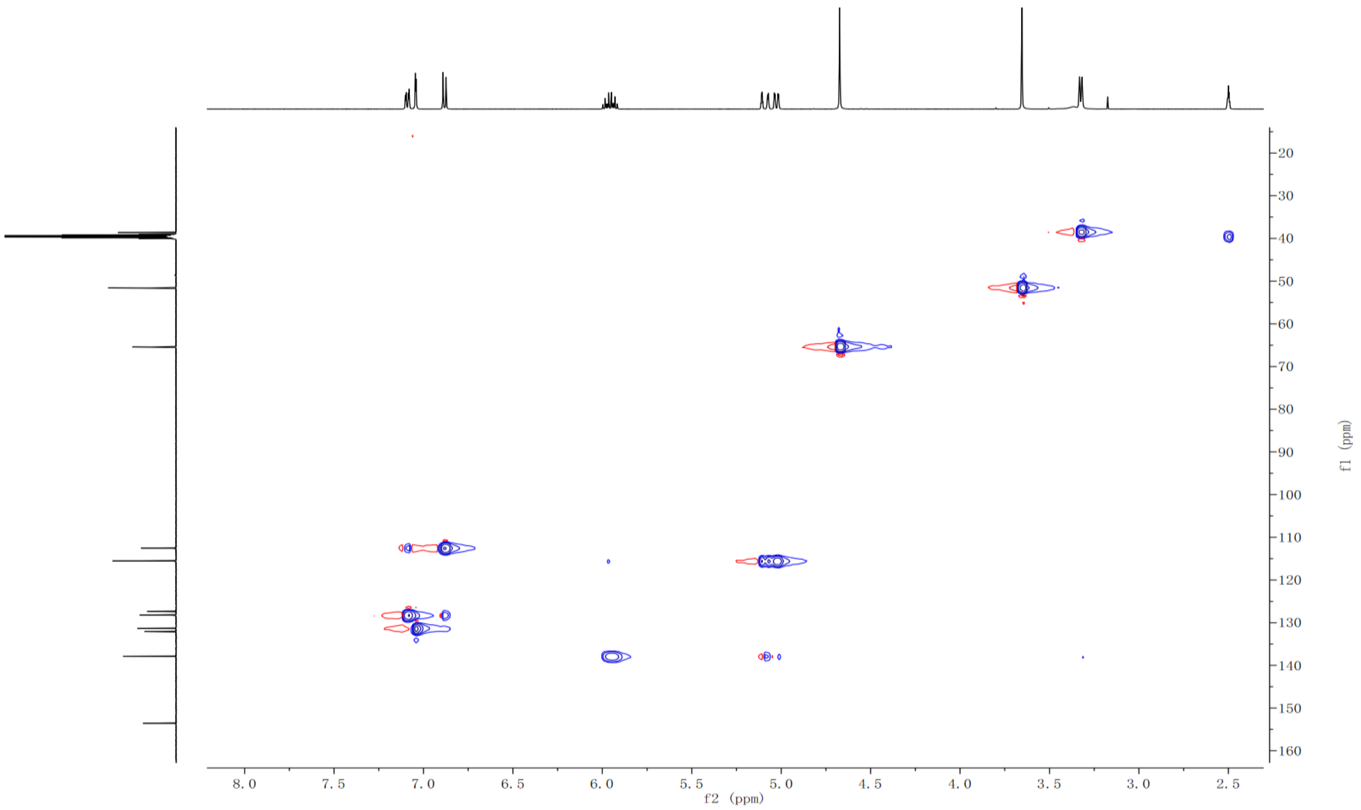

Figure S28. HMBC spectrum of **7b** in DMSO-d_6

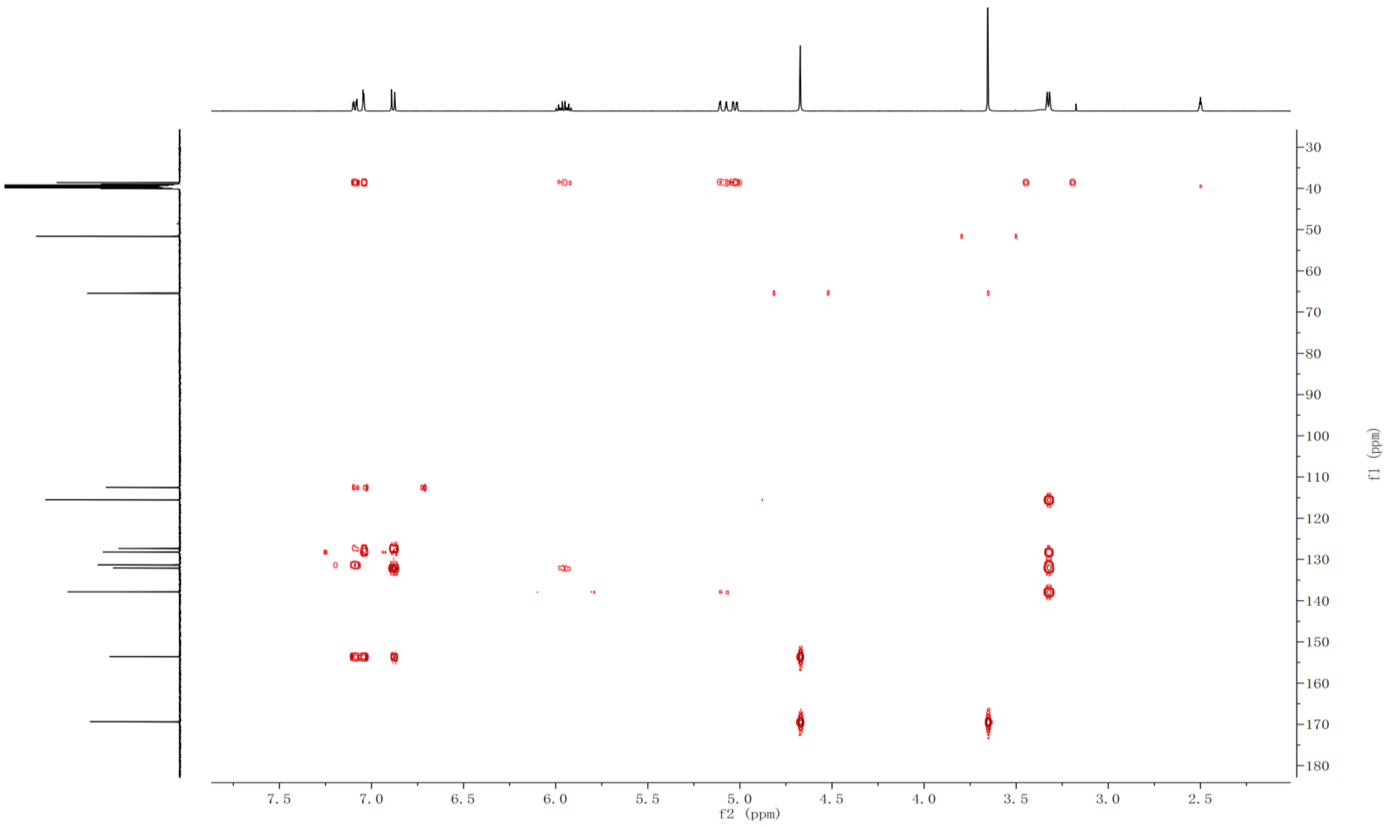

Figure S29. ^{1}H-^{1}H COSY spectrum of **3** in DMSO-d_6

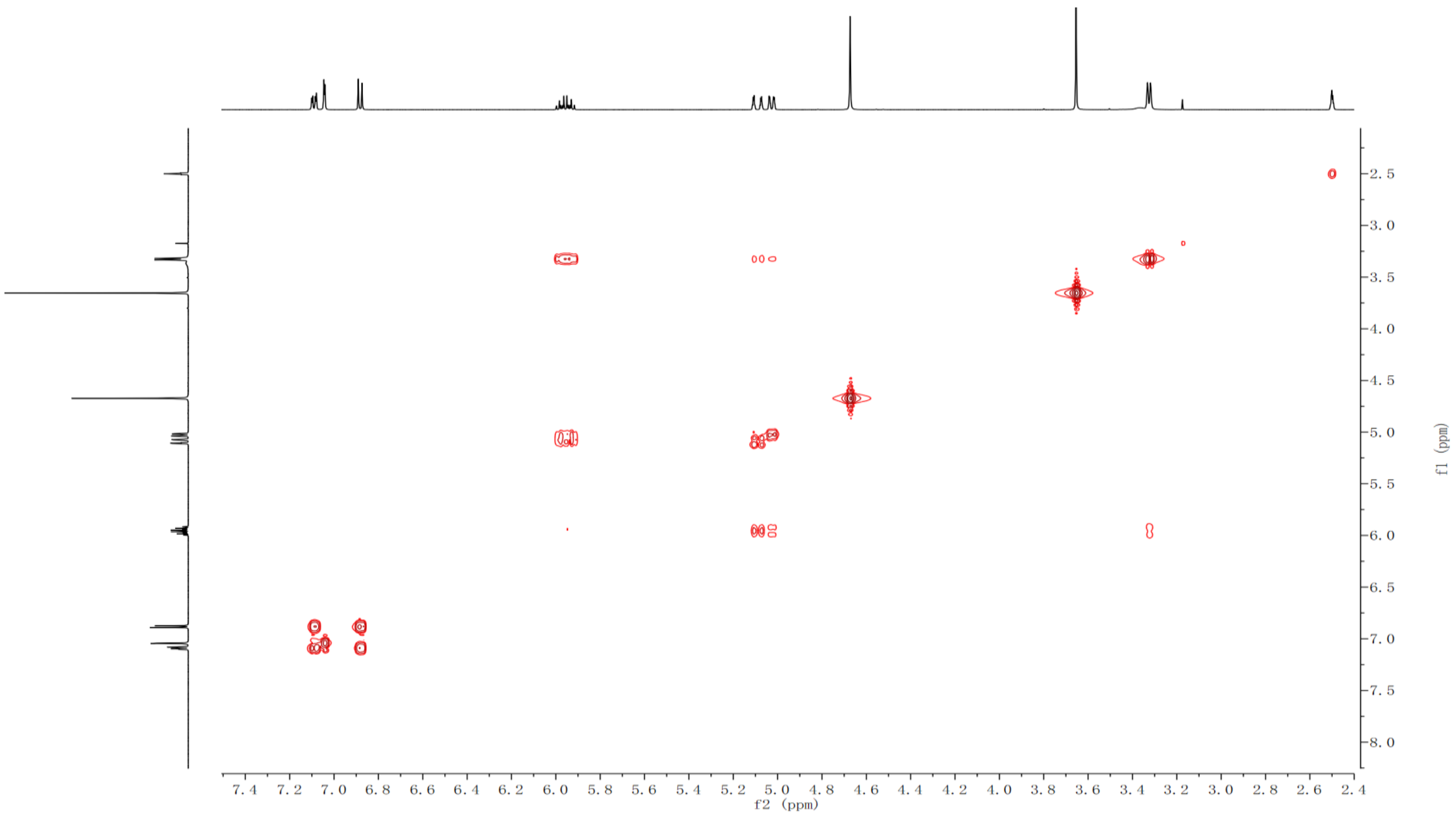

Figure S30. ^{1}H NMR (500 MHz, DMSO-d_6) spectrum of **10**

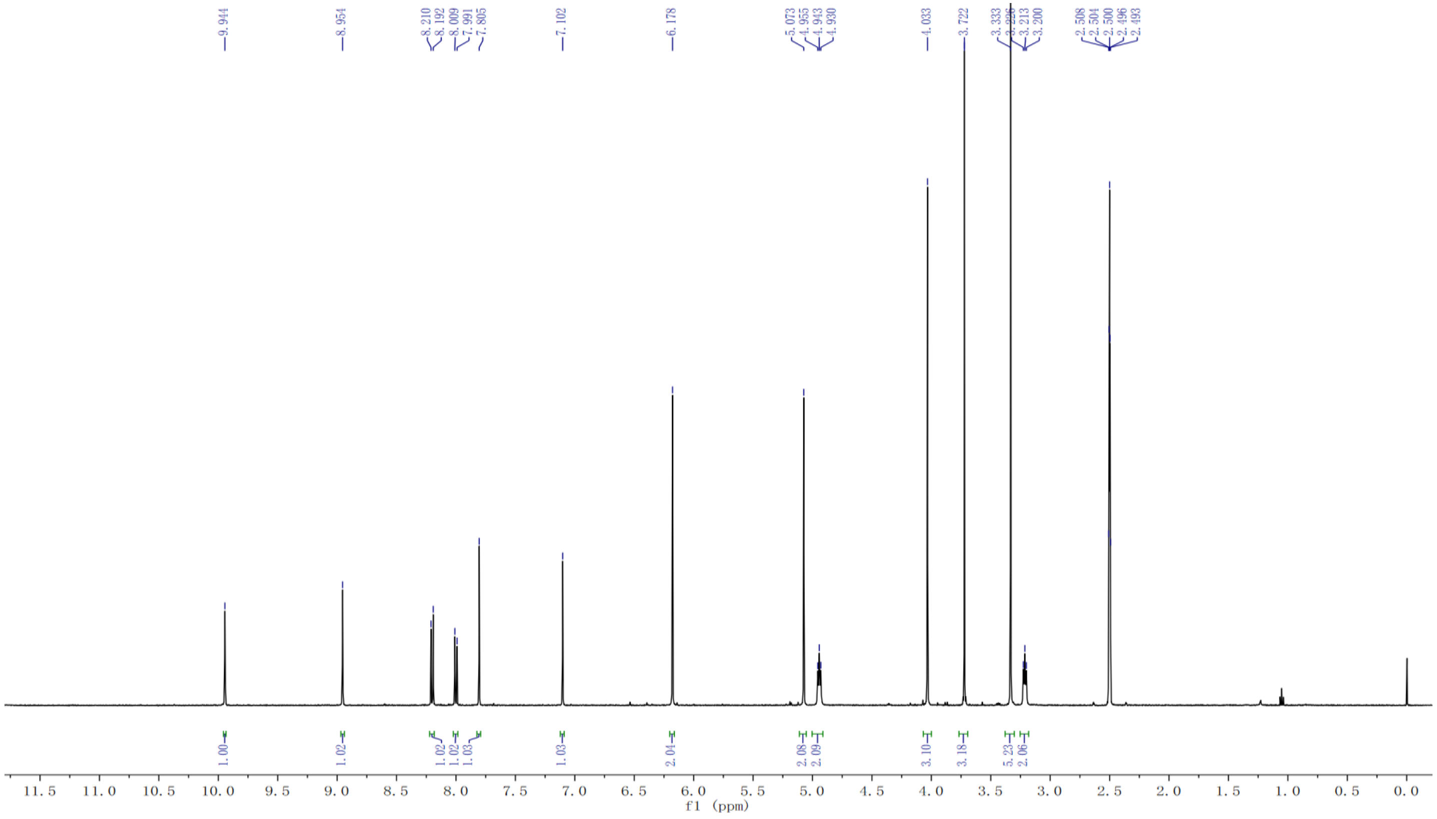

Figure S31. ^{13}C NMR (125 MHz, DMSO-d_6) spectrum of **10**

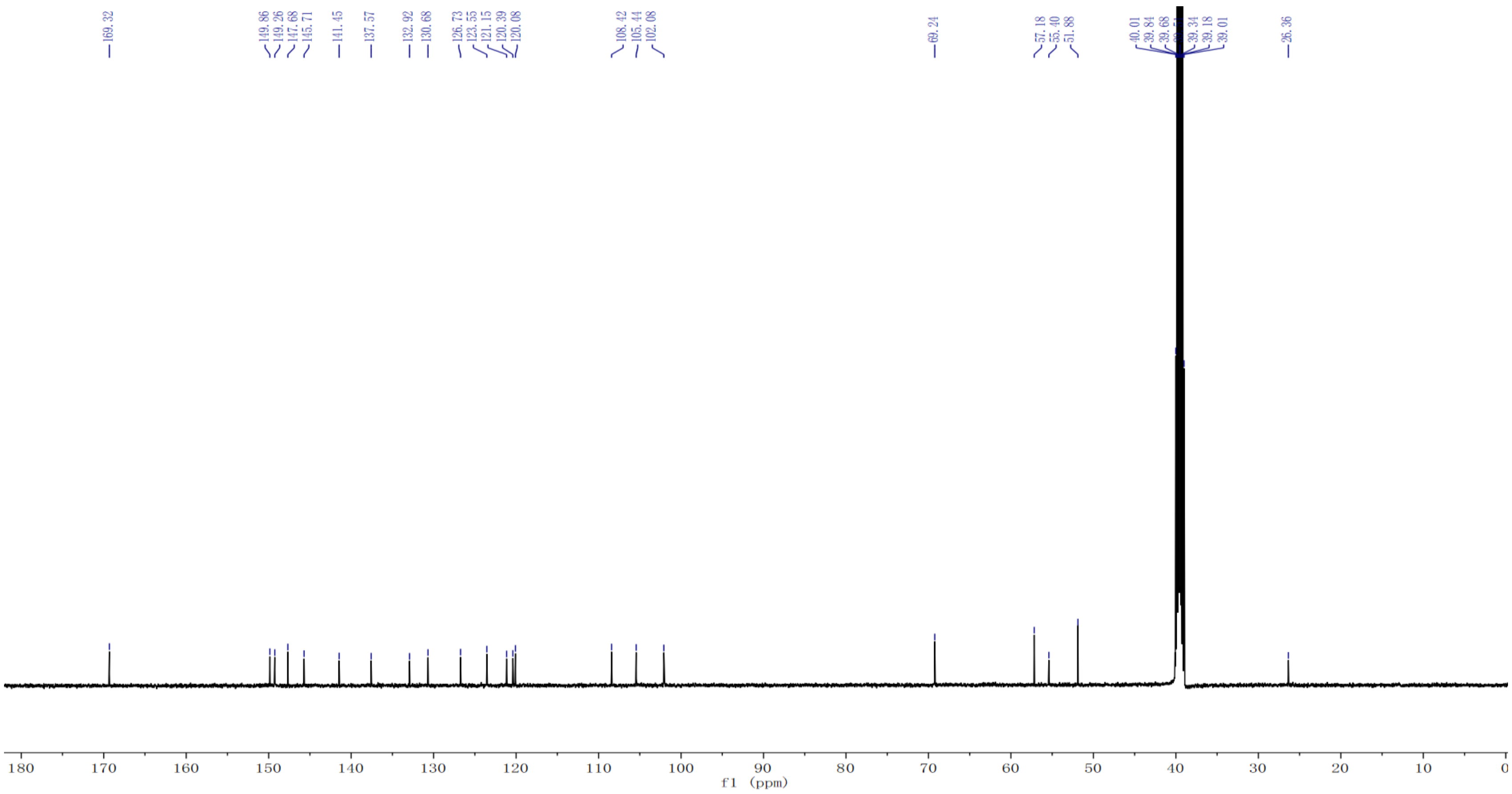

Figure S31.HSQC spectrum of **10** in DMSO-d_6

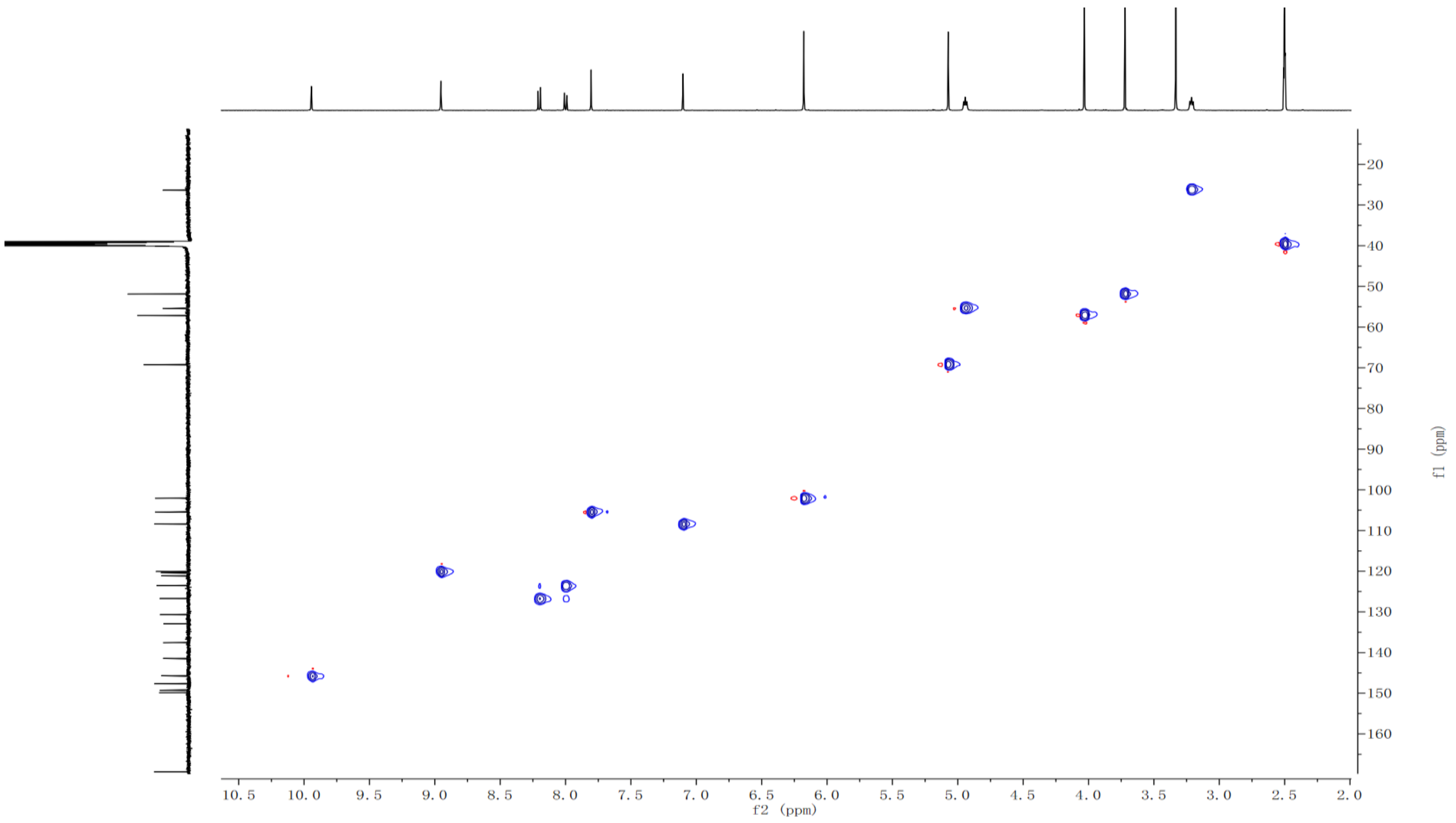

Figure S32. HMBC spectrum of **10** in DMSO-d_6

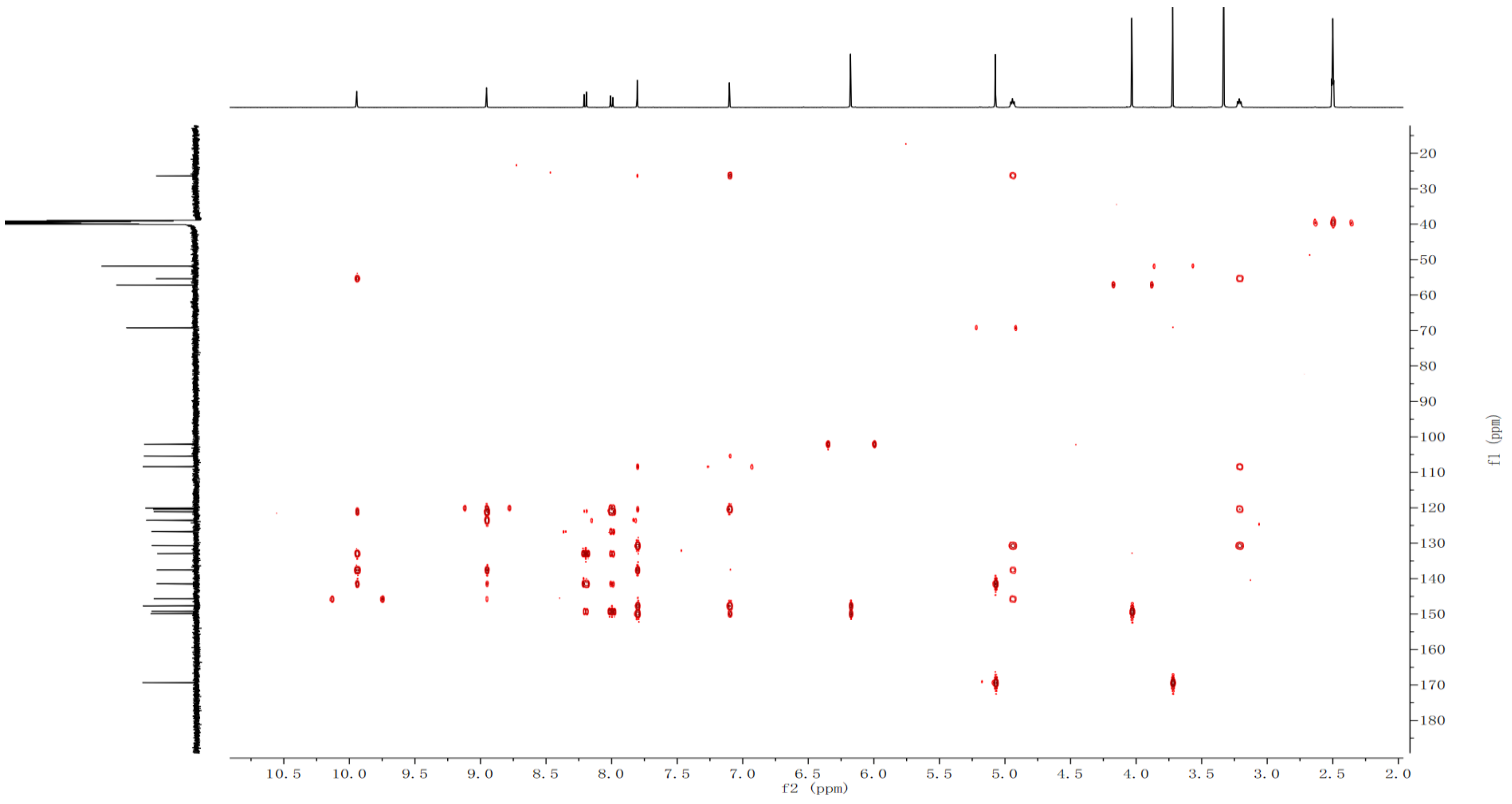

Figure S33. ^1H-^1H COSY spectrum of **10** in DMSO-d_6

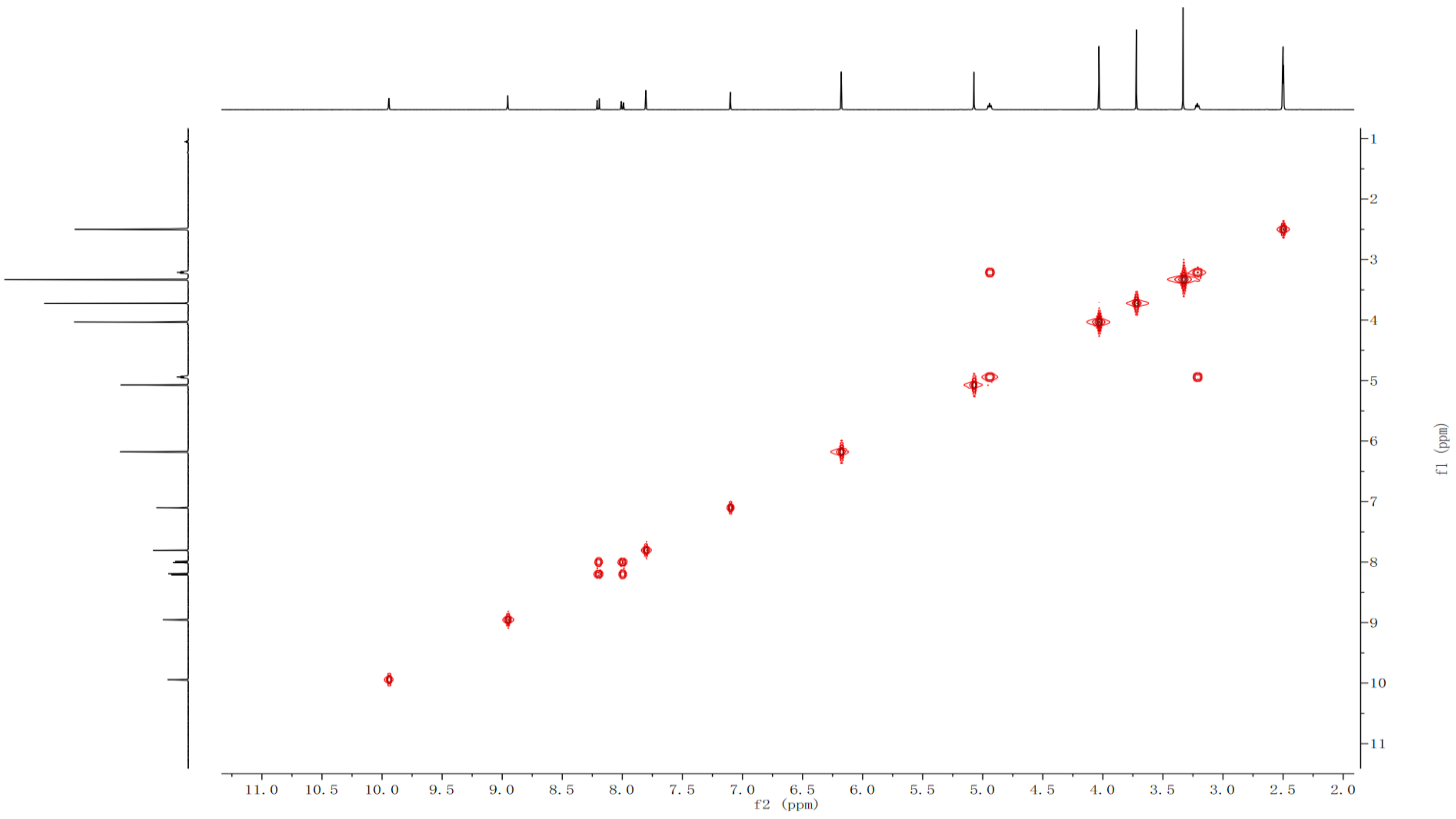

Figure S34. ^1H NMR (500 MHz, DMSO-d_6) spectrum of **9**

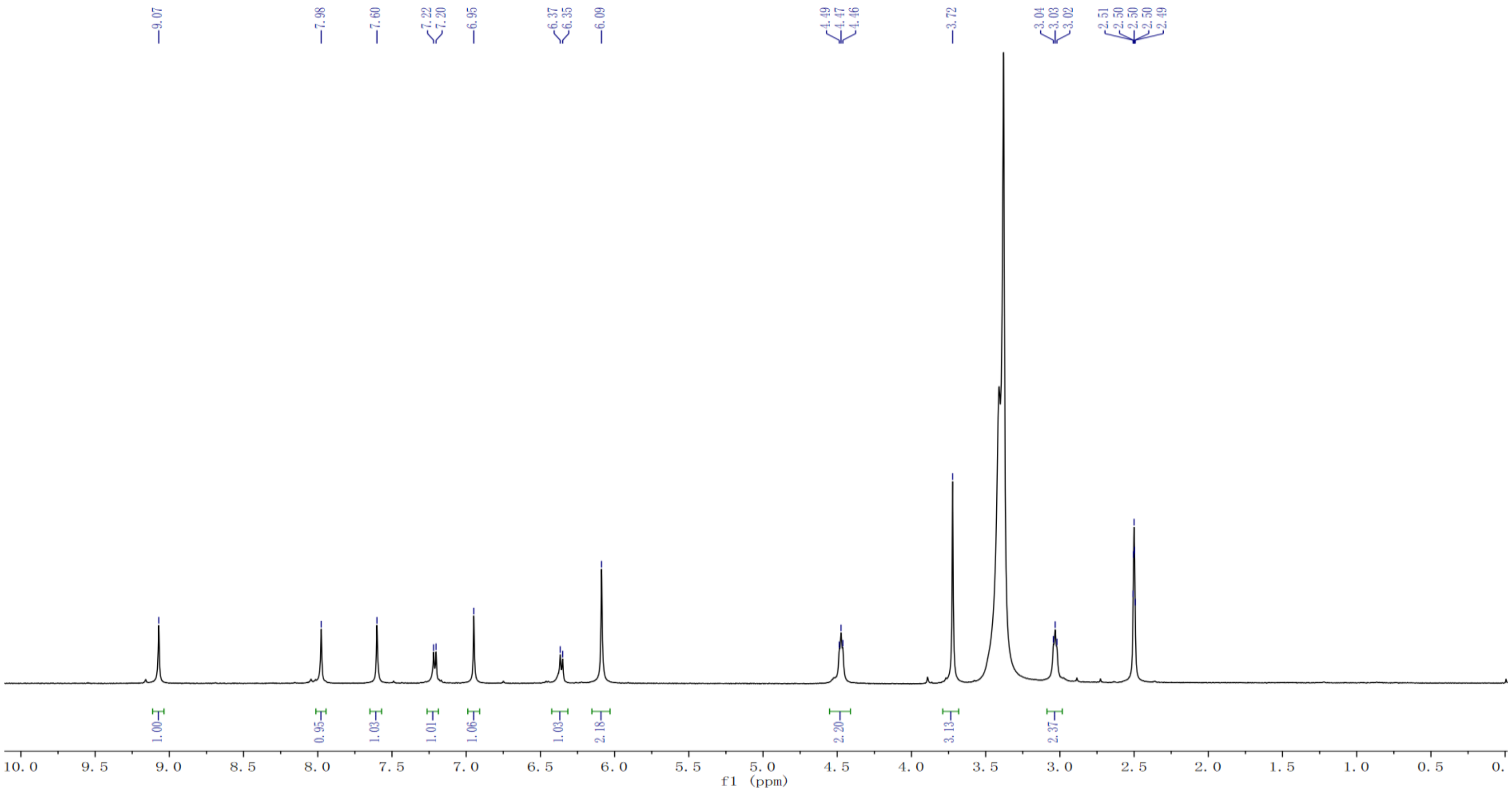

Figure S35.^{13}C NMR (125 MHz, DMSO-*d*6) spectrum of **9**

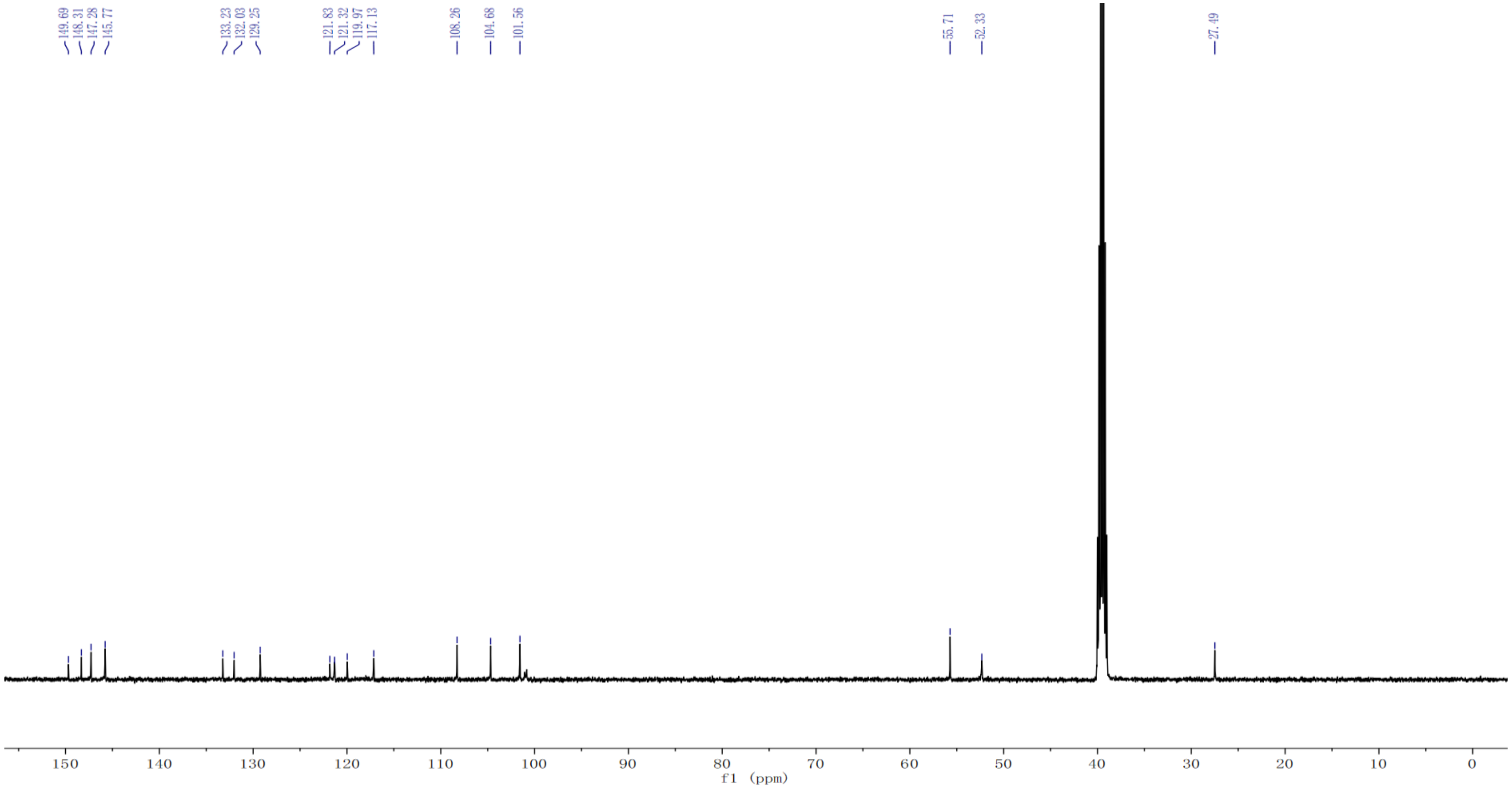

Figure S36. ^{1}H-^{1}H COSY spectrum of **9** in DMSO-*d*$_6$

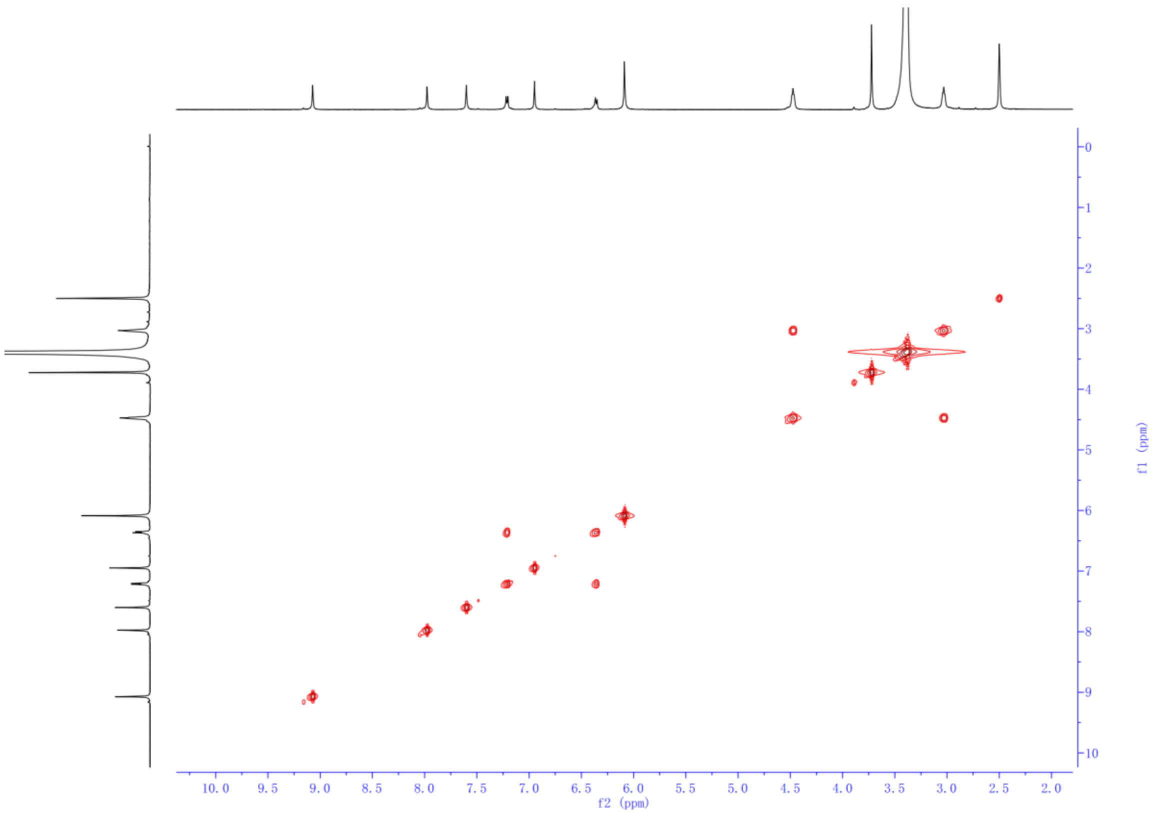

Figure S37.HSQC spectrum of **9** in DMSO-*d*$_6$

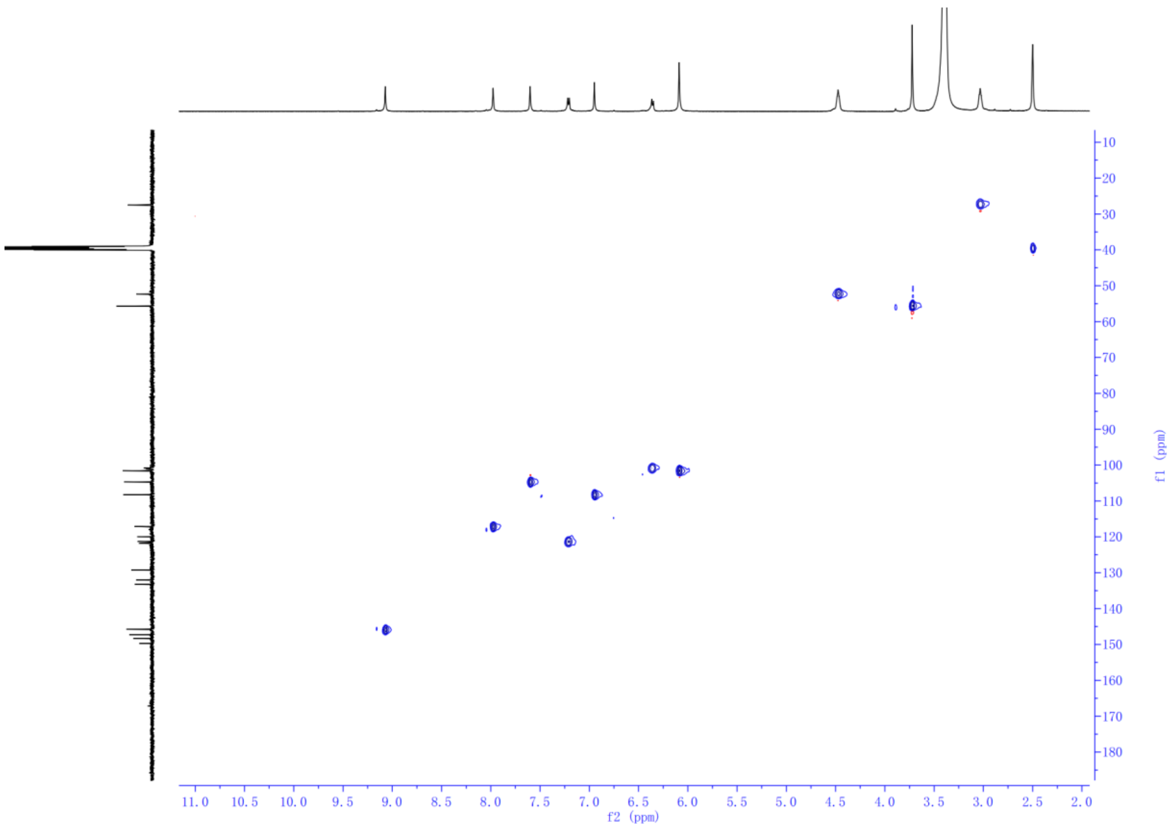

Figure S38. HMBC spectrum of **9** in DMSO-*d*₆

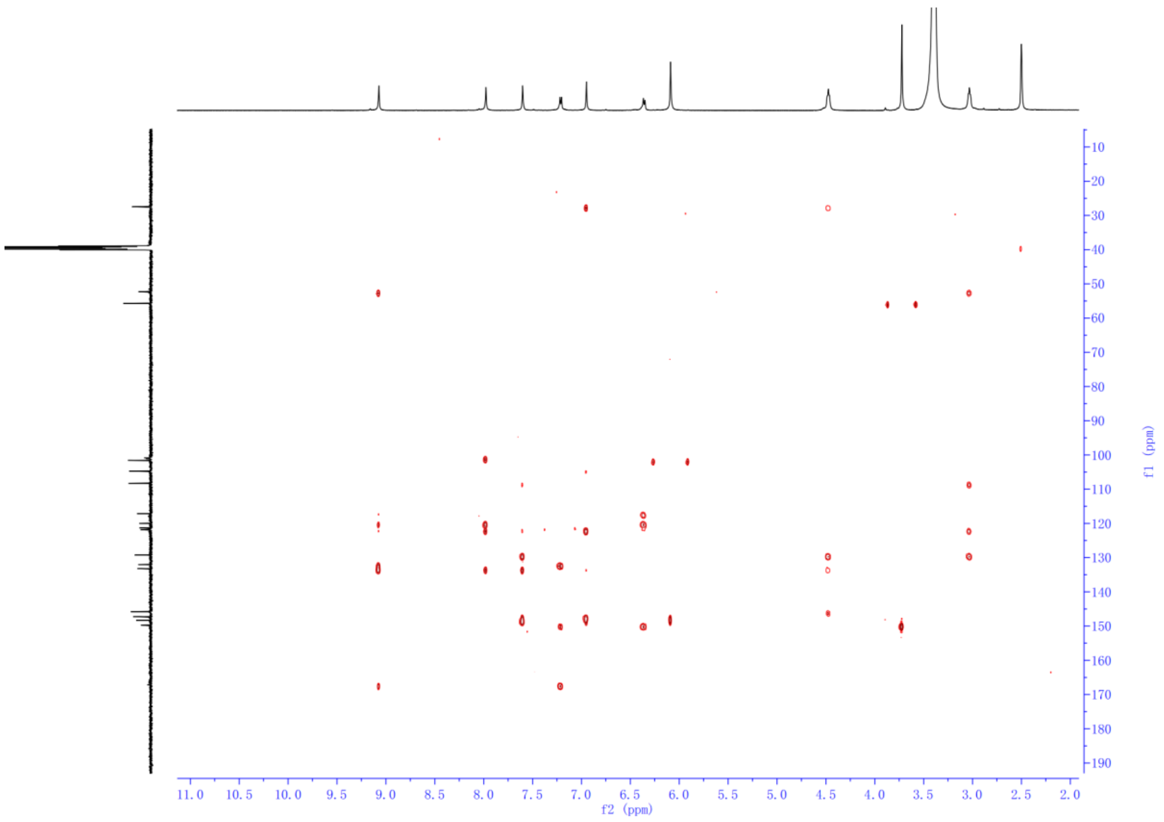

Supplement: Supplementary file 1 [file molecules-22-01752-s001.pdf]
